# Supplementary material for: Assessing the aesthetic attractivity of European butterflies: A web-based survey protocol
Source: PLoS One. 2023 May 11;18(5):e0283360. doi: 10.1371/journal.pone.0283360 (PMC10174575; doi:10.1371/journal.pone.0283360)
Supplement: S1 Table — The list of butterfly species along with the collected pictures used in section n.1 of “Unveiling” test. (DOCX) [file pone.0283360.s001.docx]

**S2 Table**

In this Table the list of butterfly species used in the test along with the 2288 collected pictures is provided. For each species is indicated with an x the presence of a photo where the butterfly is as male/female (M/F in the first letter of the acronym), in dorsal/ventral position (D/V in the second letter of the acronym), with a neutral background/background with flower (B/F in the third letter of the acronym).

| Species number | Species name | Dimorphism | MDF | MDB | MVF | MVB | FDF | FDB | FVF | FVB |
| --- | --- | --- | --- | --- | --- | --- | --- | --- | --- | --- |
| 1 | *Iphiclides podalirius* | no | x | x | x | x |  |  |  |  |
| 2 | *Iphiclides feisthamelii* | no | x | x | x | x |  |  |  |  |
| 3 | *Papilio alexanor* | no | x | x | x | x |  |  |  |  |
| 4 | *Papilio machaon* | no | x | x | x | x |  |  |  |  |
| 5 | *Papilio hospiton* | no | x | x | x | x |  |  |  |  |
| 6 | *Parnassius mnemosyne* | no | x | x | x | x |  |  |  |  |
| 7 | *Parnassius phoebus* | yes | x | x | x | x | x | x | x | x |
| 8 | *Parnassius apollo* | yes | x | x | x | x | x | x | x | x |
| 9 | *Archon apollinus* | no | x | x | x | x |  |  |  |  |
| 10 | *Zerynthia cerisy* | no | x | x | x | x |  |  |  |  |
| 11 | *Zerynthia cretica* | no | x | x | x | x |  |  |  |  |
| 12 | *Zerynthia caucasica* | no |  | x | x | x |  |  |  |  |
| 13 | *Zerynthia rumina* | no | x | x | x | x |  |  |  |  |
| 14 | *Zerynthia polyxena* | no | x | x | x | x |  |  |  |  |
| 15 | *Zerynthia cassandra* | no | x | x | x | x |  |  |  |  |
| 16 | *Heteropterus morpheus* | no | x | x | x | x |  |  |  |  |
| 17 | *Carterocephalus silvicola* | no | x | x | x | x |  |  |  |  |
| 18 | *Carterocephalus palaemon* | no | x | x | x | x |  |  |  |  |
| 19 | *Pelopidas thrax* | no | x | x | x | x |  |  |  |  |
| 20 | *Borbo borbonica* | no | x | x | x | x |  |  |  |  |
| 21 | *Gegenes pumilio* | yes | x | x | x | x | x | x |  |  |
| 22 | *Gegenes nostrodamus* | yes | x | x | x | x | x | x |  |  |
| 23 | *Ochlodes sylvanus* | yes | x | x | x | x | x | x | x | x |
| 24 | *Hesperia comma* | yes | x | x | x | x | x | x | x | x |
| 25 | *Thymelicus christi* | yes | x | x | x | x | x | x | x | x |
| 26 | *Thymelicus acteon* | yes | x | x | x | x | x | x | x | x |
| 27 | *Thymelicus hyrax* | yes | x | x | x | x | x | x | x | x |
| 28 | *Thymelicus sylvestris* | yes | x | x | x | x | x | x | x | x |
| 29 | *Thymelicus lineola* | yes | x | x | x | x | x | x | x | x |
| 30 | *Spialia phlomidis* | no | x | x | x | x |  |  |  |  |
| 31 | *Spialia sertorius* | no | x | x | x | x |  |  |  |  |
| 32 | *Spialia therapne* | no | x | x | x | x |  |  |  |  |
| 33 | *Spialia rosae* | no | x | x | x | x |  |  |  |  |
| 34 | *Spialia orbifer* | no | x | x | x | x |  |  |  |  |
| 35 | *Carcharodus tripolinus* | no | x | x | x | x |  |  |  |  |
| 36 | *Carcharodus alceae* | no | x | x | x | x |  |  |  |  |
| 37 | *Muschampia cribrellum* | no | x | x | x | x |  |  |  |  |
| 38 | *Muschampia tessellum* | no | x | x | x | x |  |  |  |  |
| 39 | *Muschampia proto* | no | x | x | x | x |  |  |  |  |
| 40 | *Carcharodus lavatherae* | no | x | x | x | x |  |  |  |  |
| 41 | *Carcharodus orientalis* | no | x | x | x | x |  |  |  |  |
| 42 | *Carcharodus floccifera* | no | x | x | x | x |  |  |  |  |
| 43 | *Carcharodus stauderi* | no | x | x | x | x |  |  |  |  |
| 44 | *Carcharodus baeticus* | no | x | x | x | x |  |  |  |  |
| 45 | *Erynnis tages* | no | x | x | x | x |  |  |  |  |
| 46 | *Erynnis marloyi* | no | x | x | x | x |  |  |  |  |
| 47 | *Pyrgus malvoides* | no | x | x | x | x |  |  |  |  |
| 48 | *Pyrgus malvae* | no | x | x | x | x |  |  |  |  |
| 49 | *Pyrgus carthami* | no | x | x | x | x |  |  |  |  |
| 50 | *Pyrgus sidae* | no | x | x | x | x |  |  |  |  |
| 51 | *Pyrgus centaureae* | no | x | x | x | x |  |  |  |  |
| 52 | *Pyrgus cacaliae* | no | x | x | x | x |  |  |  |  |
| 53 | *Pyrgus andromedae* | no | x | x | x | x |  |  |  |  |
| 54 | *Pyrgus serratulae* | no | x | x | x | x |  |  |  |  |
| 55 | *Pyrgus armoricanus* | no | x | x | x | x |  |  |  |  |
| 56 | *Pyrgus alveus* | no | x | x | x | x |  |  |  |  |
| 57 | *Pyrgus warrenensis* | no | x | x | x | x |  |  |  |  |
| 58 | *Pyrgus foulquieri* | no | x | x | x | x |  |  |  |  |
| 59 | *Pyrgus onopordi* | no | x | x | x | x |  |  |  |  |
| 60 | *Pyrgus carlinae* | no | x | x | x | x |  |  |  |  |
| 61 | *Pyrgus cirsii* | no | x | x | x | x |  |  |  |  |
| 62 | *Pyrgus cinarae* | no | x | x | x | x |  |  |  |  |
| 63 | *Leptidea duponcheli* | yes |  |  | x | x |  |  | x | x |
| 64 | *Leptidea morsei* | yes |  |  | x | x |  |  | x |  |
| 65 | *Leptidea juvernica* | yes |  |  | x | x |  |  | x | x |
| 66 | *Leptidea sinapis* | yes | x | x | x | x |  |  |  |  |
| 67 | *Leptidea reali* | yes |  |  | x | x |  |  |  |  |
| 68 | *Gonepteryx rhamni* | yes | x | x | x | x |  |  | x | x |
| 69 | *Gonepteryx cleobule* | yes | x |  | x | x |  |  | x | x |
| 70 | *Gonepteryx cleopatra* | yes | x | x | x | x | x | x | x | x |
| 71 | *Gonepteryx maderensis* | yes |  |  | x | x |  |  | x |  |
| 72 | *Gonepteryx farinosa* | yes |  | x | x | x |  |  |  |  |
| 73 | *Catopsilia florella* | yes | x |  | x | x | x | x | x | x |
| 74 | *Colias hyale* | yes | x | x | x | x | x |  | x | x |
| 75 | *Colias alfacariensis* | yes | x |  | x | x | x | x | x | x |
| 76 | *Colias phicomone* | yes | x |  | x | x |  | x |  |  |
| 77 | *Colias aurorina* | yes | x | x | x | x |  | x | x | x |
| 78 | *Colias chrysotheme* | yes |  |  | x | x |  |  | x | x |
| 79 | *Colias erate* | yes | x | x | x | x |  | x | x | x |
| 80 | *Colias crocea* | yes | x | x | x | x | x | x | x | x |
| 81 | *Colias myrmidone* | yes | x | x | x | x |  | x |  |  |
| 82 | *Colias caucasica* | yes |  |  | x | x | x |  |  |  |
| 83 | *Colias palaeno* | yes | x | x | x | x | x | x | x | x |
| 84 | *Colias tyche* | yes |  |  | x | x |  |  | x | x |
| 85 | *Colias hecla* | yes |  | x | x | x |  |  | x |  |
| 86 | *Colotis evagore* | yes | x | x | x | x | x | x | x | x |
| 87 | *Aporia crataegi* | no | x | x | x | x | x | x | x | x |
| 88 | *Pontia chloridice* | yes | x | x | x | x |  |  | x | x |
| 89 | *Pontia callidice* | yes | x | x | x | x | x | x | x | x |
| 90 | *Pontia edusa* | yes | x | x | x | x | x | x | x | x |
| 91 | *Pontia daplidice* | yes | x | x | x | x | x | x | x | x |
| 92 | *Pieris krueperi* | yes | x | x | x | x | x | x | x | x |
| 93 | *Pieris brassicae* | yes | x | x | x | x | x | x | x |  |
| 94 | *Pieris wollastoni* | yes |  |  |  |  |  |  |  |  |
| 95 | *Pieris cheiranthi* | yes |  | x | x | x | x | x | x |  |
| 96 | *Pieris rapae* | yes | x | x | x | x | x | x | x | x |
| 97 | *Pieris mannii* | yes | x | x | x | x | x | x | x | x |
| 98 | *Pieris ergane* | yes | x | x | x | x | x | x | x | x |
| 99 | *Pieris bryoniae* | yes | x | x | x | x | x | x | x | x |
| 100 | *Pieris napi* | yes | x | x | x | x | x | x | x | x |
| 101 | *Pieris balcana* | yes | x | x | x | x | x | x | x |  |
| 102 | *Euchloe tagis* | no | x | x | x | x |  |  |  |  |
| 103 | *Euchloe eversi* | no | x | x | x | x |  |  |  |  |
| 104 | *Euchloe grancanariensis* | no | x | x | x | x |  |  |  |  |
| 105 | *Euchloe hesperidum* | no | x | x | x | x |  |  |  |  |
| 106 | *Euchloe belemia* | no | x | x | x | x |  |  |  |  |
| 107 | *Euchloe insularis* | no | x | x | x | x |  |  |  |  |
| 108 | *Euchloe crameri* | no | x | x | x | x |  |  |  |  |
| 109 | *Euchloe simplonia* | no | x | x | x | x |  |  |  |  |
| 110 | *Euchloe ausonia* | no | x | x | x | x |  |  |  |  |
| 111 | *Euchloe charlonia* | no | x | x | x | x |  |  |  |  |
| 112 | *Euchloe penia* | no | x | x | x | x |  |  |  |  |
| 113 | *Euchloe bazae* | no |  | x | x | x |  |  |  |  |
| 114 | *Zegris pyrothoe* | no |  |  | x | x |  |  |  |  |
| 115 | *Zegris eupheme* | yes | x | x | x |  | x |  | x |  |
| 116 | *Anthocharis euphenoides* | yes | x | x | x | x | x | x | x | x |
| 117 | *Anthocharis cardamines* | yes | x | x | x | x | x | x | x | x |
| 118 | *Anthocharis gruneri* | yes | x | x | x | x | x | x | x |  |
| 119 | *Anthocharis damone* | yes | x | x | x | x | x |  | x | x |
| 120 | *Hamearis lucina* | no | x | x | x | x |  |  |  |  |
| 121 | *Lycaena dimorpha* | yes |  |  |  |  |  |  |  |  |
| 122 | *Lycaena helle* | yes | x | x | x | x | x | x | x | x |
| 123 | *Lycaena alciphron* | yes | x | x | x | x | x | x | x | x |
| 124 | *Lycaena thetis* | yes | x | x | x | x | x | x |  |  |
| 125 | *Lycaena thersamon* | yes | x | x | x | x | x | x |  |  |
| 126 | *Lycaena dispar* | yes | x | x | x | x | x | x | x | x |
| 127 | *Lycaena hippothoe* | yes | x | x | x | x | x | x | x | x |
| 128 | *Lycaena candens* | yes | x | x | x | x | x | x | x | x |
| 129 | *Lycaena ottomanus* | yes | x | x | x | x | x | x | x | x |
| 130 | *Lycaena bleusei* | yes | x | x | x | x | x | x |  |  |
| 131 | *Lycaena phlaeas* | no | x | x | x | x |  |  |  |  |
| 132 | *Lycaena virgaureae* | yes | x | x | x | x | x | x | x | x |
| 133 | *Lycaena tityrus* | yes | x | x | x | x | x | x | x | x |
| 134 | *Cigaritis acamas* | no |  |  | x | x |  |  |  |  |
| 135 | *Thecla betulae* | yes |  | x | x | x | x | x | x | x |
| 136 | *Favonius quercus* | yes |  | x | x | x |  | x |  |  |
| 137 | *Laeosopis roboris* | yes | x | x | x | x |  | x | x | x |
| 138 | *Tomares ballus* | yes |  |  | x | x |  |  | x | x |
| 139 | *Tomares nogelii* | yes |  |  | x | x |  |  |  |  |
| 140 | *Tomares callimachus* | yes |  |  | x | x |  |  |  |  |
| 141 | *Callophrys avis* | no |  |  | x | x |  |  |  |  |
| 142 | *Callophrys suaveola* | no |  |  |  | x |  |  |  |  |
| 143 | *Callophrys rubi* | no |  | x | x | x |  |  |  |  |
| 144 | *Callophrys chalybeitincta* | no |  |  | x | x |  |  |  |  |
| 145 | *Neolycaena rhymnus* | no |  |  | x | x |  |  |  |  |
| 146 | *Satyrium pruni* | no |  |  | x | x | x | x | x | x |
| 147 | *Satyrium ilicis* | yes |  |  | x | x | x |  | x | x |
| 148 | *Satyrium esculi* | no |  | x | x | x |  | x | x | x |
| 149 | *Satyrium ledereri* | no |  |  | x | x |  |  |  |  |
| 150 | *Satyrium w-album* | no |  |  | x | x |  |  | x | x |
| 151 | *Satyrium spini* | no | x |  | x | x | x |  |  |  |
| 152 | *Satyrium acaciae* | no |  |  | x | x |  |  | x | x |
| 153 | *Leptotes pirithous* | no | x | x | x | x |  |  |  |  |
| 154 | *Cyclyrius webbianus* | yes | x | x | x | x | x | x |  |  |
| 155 | *Azanus ubaldus* | yes |  | x | x | x |  | x |  |  |
| 156 | *Azanus jesous* | yes |  | x | x | x | x | x |  |  |
| 157 | *Lampides boeticus* | no | x | x | x | x |  |  |  |  |
| 158 | *Cacyreus marshalli* | no | x | x | x | x |  |  |  |  |
| 159 | *Celastrina argiolus* | yes | x | x | x | x | x | x |  |  |
| 160 | *Tarucus theophrastus* | no | x | x | x | x |  |  |  |  |
| 161 | *Tarucus balkanicus* | no | x | x | x | x |  |  |  |  |
| 162 | *Phengaris alcon* | yes | x | x | x | x | x | x |  |  |
| 163 | *Phengaris arion* | yes | x | x | x | x | x | x |  |  |
| 164 | *Phengaris teleius* | yes | x | x | x | x | x | x |  |  |
| 165 | *Phengaris nausithous* | yes | x | x | x | x |  | x |  |  |
| 166 | *Turanana taygetica* | yes |  | x | x | x | x |  |  |  |
| 167 | *Pseudophilotes bavius* | yes | x | x | x | x | x | x |  |  |
| 168 | *Pseudophilotes barbagiae* | no | x | x | x | x |  |  |  |  |
| 169 | *Pseudophilotes abencerragus* | yes | x | x | x | x |  | x |  |  |
| 170 | *Pseudophilotes panoptes* | yes | x | x | x | x | x |  |  |  |
| 171 | *Pseudophilotes vicrama* | yes | x | x | x | x | x | x |  |  |
| 172 | *Pseudophilotes baton* | yes | x | x | x | x | x | x |  |  |
| 173 | *Scolitantides orion* | yes | x | x | x | x | x | x |  |  |
| 174 | *Praephilotes anthracias* | no |  |  |  | x |  |  |  |  |
| 175 | *Iolana iolas* | yes | x | x | x | x | x | x |  |  |
| 176 | *Iolana debilitata* | yes |  |  | x | x |  |  |  |  |
| 177 | *Glaucopsyche melanops* | yes | x | x | x | x | x | x |  |  |
| 178 | *Glaucopsyche paphos* | yes | x | x | x | x | x | x |  |  |
| 179 | *Glaucopsyche alexis* | yes | x | x | x | x | x | x |  |  |
| 180 | *Zizeeria knysna* | yes | x | x | x | x | x | x |  |  |
| 181 | *Zizeeria karsandra* | yes | x | x | x | x | x | x |  |  |
| 182 | *Tongeia fischeri* | yes |  |  | x | x | x | x |  |  |
| 183 | *Cupido argiades* | yes | x | x | x | x | x | x |  |  |
| 184 | *Cupido decoloratus* | yes |  | x | x | x |  | x |  |  |
| 185 | *Cupido alcetas* | yes | x | x | x | x | x | x |  |  |
| 186 | *Cupido osiris* | yes | x | x | x | x | x | x |  |  |
| 187 | *Cupido minimus* | no | x | x | x | x |  |  |  |  |
| 188 | *Cupido lorquinii* | yes | x | x | x | x | x |  |  |  |
| 189 | *Luthrodes galba* | no |  |  |  | x |  |  |  |  |
| 190 | *Freyeria trochylus* | yes | x | x | x | x | x | x |  |  |
| 191 | *Plebejus argus* | yes | x | x | x | x | x | x | x | x |
| 192 | *Plebejus idas* | yes | x | x | x | x | x | x |  |  |
| 193 | *Plebejus bellieri* | yes | x | x | x | x | x | x |  |  |
| 194 | *Plebejus argyrognomon* | yes | x | x | x | x | x | x | x | x |
| 195 | *Agriades orbitulus* | yes | x | x | x | x | x | x |  |  |
| 196 | *Agriades optilete* | yes | x | x | x | x |  | x |  |  |
| 197 | *Agriades pyrenaicus* | yes | x | x | x | x | x | x |  |  |
| 198 | *Agriades dardanus* | yes | x | x | x | x | x | x |  |  |
| 199 | *Agriades zullichi* | yes | x | x |  | x |  | x |  |  |
| 200 | *Agriades glandon* | yes | x | x | x | x | x | x |  |  |
| 201 | *Agriades aquilo* | yes |  | x | x | x |  | x |  |  |
| 202 | *Plebejidea loewii* | yes |  | x | x | x | x | x |  |  |
| 203 | *Eumedonia eumedon* | no | x | x | x | x |  |  |  |  |
| 204 | *Kretania psylorita* | yes |  | x | x | x |  | x |  |  |
| 205 | *Kretania hespericus* | yes |  |  |  |  |  |  |  |  |
| 206 | *Kretania eurypilus* | yes |  | x | x | x |  | x |  |  |
| 207 | *Kretania trappi* | yes | x | x | x | x |  |  |  |  |
| 208 | *Kretania sephirus* | yes |  | x | x | x |  | x |  |  |
| 209 | *Kretania pylaon* | yes |  | x | x | x |  | x |  |  |
| 210 | *Cyaniris semiargus* | yes | x | x | x | x | x | x |  |  |
| 211 | *Glabroculus cyane* | yes | x |  | x | x |  |  |  |  |
| 212 | *Aricia morronensis* | no | x | x | x | x | x | x |  |  |
| 213 | *Aricia anteros* | yes | x | x | x | x | x | x |  |  |
| 214 | *Aricia cramera* | no | x | x | x | x |  |  |  |  |
| 215 | *Aricia nicias* | yes | x | x | x | x | x | x |  |  |
| 216 | *Aricia artaxerxes* | yes | x | x | x | x | x | x |  |  |
| 217 | *Aricia montensis* | no | x | x | x | x |  |  |  |  |
| 218 | *Aricia agestis* | no | x | x | x | x |  |  |  |  |
| 219 | *Neolysandra coelestina* | yes | x | x | x | x | x | x | x | x |
| 220 | *Lysandra hispana* | yes | x | x | x | x | x | x | x | x |
| 221 | *Lysandra corydonius* | yes |  | x |  | x |  |  |  |  |
| 222 | *Lysandra bellargus* | yes | x | x | x | x | x | x | x | x |
| 223 | *Lysandra coridon* | yes | x | x | x | x | x | x | x | x |
| 224 | *Lysandra caelestissima* | yes | x | x | x | x | x | x | x | x |
| 225 | *Lysandra albicans* | yes | x | x | x | x |  | x | x | x |
| 226 | *Polyommatus escheri* | yes | x | x | x | x | x | x | x | x |
| 227 | *Polyommatus thersites* | yes | x | x | x | x | x | x | x | x |
| 228 | *Polyommatus daphnis* | yes | x | x | x | x | x | x | x | x |
| 229 | *Polyommatus amandus* | yes | x | x | x | x | x | x | x | x |
| 230 | *Polyommatus golgus* | yes | x | x | x | x | x | x |  |  |
| 231 | *Polyommatus nivescens* | yes | x | x | x | x | x | x | x |  |
| 232 | *Polyommatus dorylas* | yes | x | x | x | x | x | x | x | x |
| 233 | *Polyommatus celina* | yes | x | x | x | x | x | x | x | x |
| 234 | *Polyommatus icarus* | yes | x | x | x | x | x | x | x | x |
| 235 | *Polyommatus eros* | yes | x | x | x | x | x | x | x | x |
| 236 | *Polyommatus damon* | yes | x | x | x | x | x | x |  |  |
| 237 | *Polyommatus damone* | yes |  |  | x |  |  |  |  |  |
| 238 | *Polyommatus damocles* | yes |  |  | x |  |  |  |  |  |
| 239 | *Polyommatus admetus* | yes | x |  | x | x | x | x |  |  |
| 240 | *Polyommatus ripartii* | no | x | x | x | x | x | x |  |  |
| 241 | *Polyommatus nephohiptamenos* | yes | x | x | x | x | x |  |  |  |
| 242 | *Polyommatus iphigenia* | yes | x | x | x | x | x |  |  |  |
| 243 | *Polyommatus violetae* | yes | x | x | x | x | x | x |  |  |
| 244 | *Polyommatus fulgens* | no | x | x | x | x |  |  |  |  |
| 245 | *Polyommatus fabressei* | yes | x | x | x | x | x | x |  |  |
| 246 | *Polyommatus dolus* | yes | x | x | x | x | x | x | x | x |
| 247 | *Polyommatus humedasae* | no | x | x | x | x |  |  |  |  |
| 248 | *Polyommatus timfristos* | yes | x |  | x | x | x | x |  |  |
| 249 | *Polyommatus orphicus* | yes |  |  |  |  |  |  |  |  |
| 250 | *Polyommatus aroaniensis* | no | x | x | x | x |  |  |  |  |
| 251 | *Neptis sappho* | no | x | x | x | x |  |  |  |  |
| 252 | *Neptis rivularis* | no | x | x | x | x |  |  |  |  |
| 253 | *Limenitis reducta* | no | x | x | x | x |  |  |  |  |
| 254 | *Limenitis populi* | no | x | x | x | x | x | x |  | x |
| 255 | *Limenitis camilla* | no | x | x | x | x |  |  |  |  |
| 256 | *Issoria lathonia* | no | x | x | x | x | x | x |  |  |
| 257 | *Issoria eugenia* | yes |  |  |  | x | x | x |  |  |
| 258 | *Brenthis hecate* | no | x | x | x | x | x | x |  |  |
| 259 | *Brenthis ino* | no | x | x | x | x | x | x |  |  |
| 260 | *Brenthis daphne* | no | x | x | x | x |  |  |  |  |
| 261 | *Argynnis paphia* | yes | x | x | x | x | x | x |  |  |
| 262 | *Argynnis pandora* | yes | x | x | x | x | x | x | x | x |
| 263 | *Argynnis laodice* | no | x | x | x | x |  |  |  |  |
| 264 | *Speyeria aglaja* | yes | x | x | x | x | x | x |  |  |
| 265 | *Fabriciana elisa* | no | x | x | x | x |  |  |  |  |
| 266 | *Fabriciana niobe* | no | x | x | x | x |  |  |  |  |
| 267 | *Fabriciana adippe* | no | x | x | x | x |  |  |  |  |
| 268 | *Boloria eunomia* | yes | x | x | x | x | x | x |  |  |
| 269 | *Boloria graeca* | no | x | x | x | x |  |  |  |  |
| 270 | *Boloria pales* | yes | x | x | x | x | x | x |  |  |
| 271 | *Boloria alaskensis* | no | x | x | x | x |  |  |  |  |
| 272 | *Boloria napaea* | yes | x | x | x | x | x | x |  |  |
| 273 | *Boloria aquilonaris* | yes | x | x | x | x | x | x |  |  |
| 274 | *Boloria tritonia* | yes |  |  |  |  |  |  |  |  |
| 275 | *Boloria polaris* | yes |  | x |  | x |  | x |  |  |
| 276 | *Boloria thore* | yes | x | x | x | x | x | x |  |  |
| 277 | *Boloria selene* | yes | x | x | x | x | x | x |  |  |
| 278 | *Boloria euphrosyne* | no | x | x | x | x |  |  |  |  |
| 279 | *Boloria dia* | no | x | x | x | x |  |  |  |  |
| 280 | *Boloria improba* | no | x | x | x | x |  |  |  |  |
| 281 | *Boloria frigga* | no | x | x | x | x |  |  |  |  |
| 282 | *Boloria freija* | yes | x | x | x | x | x | x | x | x |
| 283 | *Boloria selenis* | yes | x | x |  | x |  | x |  |  |
| 284 | *Boloria oscarus* | no |  | x |  | x |  |  |  |  |
| 285 | *Boloria titania* | no | x | x | x | x |  |  |  |  |
| 286 | *Boloria chariclea* | no | x | x | x | x |  |  |  |  |
| 287 | *Boloria angarensis* | no | x |  | x | x |  |  |  |  |
| 288 | *Apatura iris* | yes | x | x | x | x | x | x |  |  |
| 289 | *Apatura metis* | yes | x | x |  | x |  | x |  |  |
| 290 | *Apatura ilia* | yes |  | x |  | x |  | x |  |  |
| 291 | *Araschnia levana* | no | x | x | x | x | x | x | x | x |
| 292 | *Vanessa virginiensis* | no | x | x | x | x |  |  |  |  |
| 293 | *Vanessa cardui* | no | x | x | x | x |  |  |  |  |
| 294 | *Vanessa vulcania* | no | x | x | x | x |  |  |  |  |
| 295 | *Vanessa atalanta* | no | x | x | x | x |  |  |  |  |
| 296 | *Aglais io* | no | x | x | x | x |  |  |  |  |
| 297 | *Aglais urticae* | no | x | x | x | x |  |  |  |  |
| 298 | *Aglais ichnusa* | no | x | x |  | x |  |  |  |  |
| 299 | *Polygonia egea* | no | x | x | x | x | x | x |  |  |
| 300 | *Polygonia c-album* | no | x | x | x | x | x | x |  | x |
| 301 | *Nymphalis vaualbum* | no |  | x |  | x |  |  |  |  |
| 302 | *Nymphalis polychloros* | no | x | x | x | x |  |  |  |  |
| 303 | *Nymphalis xanthomelas* | no | x | x | x | x |  |  |  |  |
| 304 | *Nymphalis antiopa* | no |  | x |  | x |  |  |  |  |
| 305 | *Hypolimnas misippus* | yes | x | x | x | x | x | x | x | x |
| 306 | *Euphydryas desfontainii* | yes | x | x | x | x | x | x |  |  |
| 307 | *Euphydryas aurinia* | yes | x | x | x | x | x | x |  |  |
| 308 | *Euphydryas cynthia* | yes | x | x | x | x | x | x | x | x |
| 309 | *Euphydryas iduna* | yes |  | x | x | x | x | x |  |  |
| 310 | *Euphydryas maturna* | no | x | x | x | x |  |  |  |  |
| 311 | *Euphydryas intermedia* | no | x | x | x | x |  |  |  |  |
| 312 | *Melitaea trivia* | yes | x | x | x | x |  |  |  |  |
| 313 | *Melitaea didyma* | yes | x | x | x | x | x | x |  |  |
| 314 | *Melitaea arduinna* | yes | x | x | x | x | x | x |  |  |
| 315 | *Melitaea aetherie* | yes | x | x | x | x | x |  |  |  |
| 316 | *Melitaea phoebe* | yes | x | x | x | x | x | x |  |  |
| 317 | *Melitaea ornata* | yes | x | x | x | x | x | x |  |  |
| 318 | *Melitaea cinxia* | yes | x | x | x | x | x | x |  |  |
| 319 | *Melitaea diamina* | yes | x | x | x | x | x | x |  |  |
| 320 | *Melitaea celadussa* | yes | x | x | x | x | x | x |  |  |
| 321 | *Melitaea deione* | yes | x | x | x | x | x | x |  |  |
| 322 | *Melitaea britomartis* | yes | x | x | x | x | x | x |  |  |
| 323 | *Melitaea athalia* | yes | x | x | x | x | x | x |  |  |
| 324 | *Melitaea varia* | yes | x | x | x | x | x | x |  |  |
| 325 | *Melitaea parthenoides* | yes | x | x | x | x | x | x |  |  |
| 326 | *Melitaea aurelia* | yes | x | x | x | x | x | x |  |  |
| 327 | *Melitaea asteria* | yes | x | x | x | x |  |  |  |  |
| 328 | *Libythea celtis* | no | x | x | x | x |  |  |  |  |
| 329 | *Danaus plexippus* | no | x | x | x | x |  |  |  |  |
| 330 | *Danaus chrysippus* | no | x | x | x | x |  |  |  |  |
| 331 | *Charaxes jasius* | yes |  | x |  | x |  | x |  | x |
| 332 | *Coenonympha phryne* | no |  |  |  |  |  |  |  |  |
| 333 | *Coenonympha oedippus* | no |  | x | x | x |  |  |  |  |
| 334 | *Coenonympha dorus* | no | x |  | x | x |  |  |  |  |
| 335 | *Coenonympha thyrsis* | no | x |  | x | x |  |  |  |  |
| 336 | *Coenonympha pamphilus* | no | x | x | x | x |  |  |  |  |
| 337 | *Coenonympha tullia* | no |  |  | x | x |  |  |  |  |
| 338 | *Coenonympha rhodopensis* | no |  | x | x | x |  |  |  |  |
| 339 | *Coenonympha amaryllis* | no |  |  | x | x |  |  |  |  |
| 340 | *Coenonympha glycerion* | no | x |  | x | x |  |  |  |  |
| 341 | *Coenonympha corinna* | yes |  | x | x | x |  |  |  |  |
| 342 | *Coenonympha leander* | no |  |  | x | x |  |  |  |  |
| 343 | *Coenonympha hero* | no | x | x | x | x |  |  |  |  |
| 344 | *Coenonympha gardetta* | no |  | x | x | x |  |  |  |  |
| 345 | *Coenonympha orientalis* | no |  |  | x | x |  |  |  |  |
| 346 | *Coenonympha arcania* | no |  | x | x | x |  |  |  |  |
| 347 | *Kirinia roxelana* | yes |  | x | x | x |  |  | x | x |
| 348 | *Kirinia climene* | yes |  | x | x | x |  | x | x | x |
| 349 | *Lopinga achine* | yes |  | x | x | x |  | x | x | x |
| 350 | *Pararge xiphia* | no | x | x | x | x |  |  |  |  |
| 351 | *Pararge xiphioides* | no | x | x | x | x |  |  |  |  |
| 352 | *Pararge aegeria* | no | x | x | x | x |  |  |  |  |
| 353 | *Lasiommata maera* | yes | x | x | x | x | x | x |  |  |
| 354 | *Lasiommata deidamia* | yes | x | x | x | x | x |  |  |  |
| 355 | *Lasiommata petropolitana* | yes | x | x | x | x | x | x |  |  |
| 356 | *Lasiommata paramegaera* | yes | x | x | x | x | x | x |  |  |
| 357 | *Lasiommata megera* | yes | x | x | x | x | x | x |  |  |
| 358 | *Melanargia russiae* | no | x | x | x | x |  |  |  |  |
| 359 | *Melanargia larissa* | no | x | x | x | x |  |  |  |  |
| 360 | *Melanargia lachesis* | no | x | x | x | x |  |  |  |  |
| 361 | *Melanargia galathea* | no | x | x | x | x |  |  |  |  |
| 362 | *Melanargia ines* | no | x | x | x | x |  |  |  |  |
| 363 | *Melanargia arge* | no | x | x | x | x |  |  |  |  |
| 364 | *Melanargia pherusa* | no | x | x | x | x |  |  |  |  |
| 365 | *Melanargia occitanica* | no | x | x | x | x |  |  |  |  |
| 366 | *Hipparchia fatua* | yes |  | x | x | x |  | x | x | x |
| 367 | *Hipparchia statilinus* | yes | x | x | x | x | x | x | x | x |
| 368 | *Hipparchia tilosi* | yes |  |  |  | x |  |  |  |  |
| 369 | *Hipparchia bacchus* | yes |  |  |  | x |  |  |  |  |
| 370 | *Hipparchia wyssii* | yes |  |  |  | x |  | x |  |  |
| 371 | *Hipparchia tamadabae* | yes |  |  |  | x |  | x |  |  |
| 372 | *Hipparchia gomera* | yes |  | x | x | x |  |  |  |  |
| 373 | *Hipparchia fidia* | yes |  | x | x | x |  | x |  | x |
| 374 | *Hipparchia neomiris* | yes | x | x | x | x | x | x | x | x |
| 375 | *Hipparchia autonoe* | yes |  | x | x | x | x |  |  |  |
| 376 | *Hipparchia hermione* | yes |  | x | x | x |  | x |  | x |
| 377 | *Hipparchia syriaca* | yes |  | x |  | x |  | x |  | x |
| 378 | *Hipparchia fagi* | yes |  | x | x | x |  | x |  | x |
| 379 | *Hipparchia mersina* | yes |  | x |  | x |  |  |  |  |
| 380 | *Hipparchia miguelensis* | yes |  |  | x | x |  |  |  |  |
| 381 | *Hipparchia azorina* | yes |  | x | x | x |  |  |  |  |
| 382 | *Hipparchia senthes* | yes |  | x | x | x |  |  |  |  |
| 383 | *Hipparchia maderensis* | yes |  |  | x | x |  | x |  |  |
| 384 | *Hipparchia semele* | yes | x | x | x | x |  | x | x | x |
| 385 | *Hipparchia blachieri* | yes |  | x | x | x |  |  |  |  |
| 386 | *Hipparchia aristaeus* | yes | x | x | x | x | x | x |  |  |
| 387 | *Hipparchia volgensis* | yes |  |  |  | x |  |  |  |  |
| 388 | *Hipparchia neapolitana* | yes |  |  |  | x |  |  |  |  |
| 389 | *Hipparchia leighebi* | yes |  |  | x | x |  |  |  |  |
| 390 | *Hipparchia pellucida* | yes |  | x | x | x |  |  |  |  |
| 391 | *Hipparchia sbordonii* | yes |  |  | x | x |  |  |  |  |
| 392 | *Hipparchia cypriensis* | yes |  |  | x | x |  | x |  | x |
| 393 | *Hipparchia cretica* | yes | x | x | x | x |  |  |  |  |
| 394 | *Hipparchia christenseni* | yes |  | x |  | x |  |  |  |  |
| 395 | *Minois dryas* | yes | x | x | x | x | x | x | x | x |
| 396 | *Brintesia circe* | no | x | x | x | x |  |  |  |  |
| 397 | *Arethusana arethusa* | no | x | x | x | x | x | x |  |  |
| 398 | *Oeneis tarpeia* | no |  |  |  | x |  |  |  |  |
| 399 | *Oeneis bore* | yes |  | x | x | x |  |  |  | x |
| 400 | *Oeneis ammon* | no |  |  |  | x |  |  |  |  |
| 401 | *Oeneis melissa* | no |  |  |  | x |  |  |  |  |
| 402 | *Oeneis magna* | no |  |  | x | x |  |  |  |  |
| 403 | *Oeneis jutta* | yes |  | x | x | x |  | x |  | x |
| 404 | *Oeneis norna* | yes |  | x | x | x |  | x |  | x |
| 405 | *Oeneis polixenes* | yes |  |  | x | x |  |  |  |  |
| 406 | *Oeneis glacialis* | yes |  | x | x | x |  | x | x | x |
| 407 | *Satyrus ferula* | yes | x | x | x | x | x | x | x | x |
| 408 | *Satyrus virbius* | yes |  |  | x | x |  |  |  | x |
| 409 | *Satyrus actaea* | yes |  | x | x | x |  | x | x | x |
| 410 | *Chazara briseis* | yes | x | x | x | x | x | x | x | x |
| 411 | *Chazara prieuri* | yes |  | x | x | x |  | x | x | x |
| 412 | *Chazara persephone* | yes |  | x | x | x |  |  | x | x |
| 413 | *Pseudochazara geyeri* | yes |  |  | x | x |  |  |  | x |
| 414 | *Pseudochazara graeca* | yes | x | x | x | x |  |  |  |  |
| 415 | *Pseudochazara amymone* | yes |  | x | x | x |  |  |  |  |
| 416 | *Pseudochazara anthelea* | yes | x | x | x | x | x |  | x | x |
| 417 | *Pseudochazara amalthea* | yes |  |  |  | x |  |  |  |  |
| 418 | *Pseudochazara williamsi* | yes |  |  |  | x |  |  |  |  |
| 419 | *Pseudochazara euxina* | yes |  |  |  | x |  |  |  |  |
| 420 | *Pseudochazara mercurius* | yes |  |  |  |  |  |  |  |  |
| 421 | *Pseudochazara cingovskii* | yes |  |  | x | x |  |  |  |  |
| 422 | *Pseudochazara tisiphone* | yes |  |  |  | x |  |  |  |  |
| 423 | *Pseudochazara orestes* | yes |  |  | x | x |  |  |  |  |
| 424 | *Ypthima asterope* | no | x | x | x | x |  |  |  |  |
| 425 | *Proterebia phegea* | yes |  | x | x | x | x | x |  | x |
| 426 | *Hyponephele huebneri* | yes |  |  |  |  |  |  |  |  |
| 427 | *Hyponephele lycaon* | yes |  | x | x | x | x | x | x | x |
| 428 | *Hyponephele lupina* | yes |  | x | x | x | x |  | x | x |
| 429 | *Aphantopus hyperantus* | yes |  | x | x | x | x | x | x | x |
| 430 | *Pyronia cecilia* | yes | x | x | x | x | x | x | x | x |
| 431 | *Pyronia tithonus* | yes | x | x | x | x | x | x | x | x |
| 432 | *Pyronia bathseba* | yes | x | x | x | x | x | x | x | x |
| 433 | *Maniola jurtina* | yes | x | x | x | x | x | x | x | x |
| 434 | *Maniola nurag* | yes | x | x | x | x | x |  |  |  |
| 435 | *Maniola chia* | yes | x | x | x | x |  | x | x | x |
| 436 | *Maniola megala* | yes |  | x |  |  |  |  |  |  |
| 437 | *Maniola cypricola* | yes |  | x | x | x | x | x | x | x |
| 438 | *Maniola telmessia* | yes | x | x | x | x | x | x | x | x |
| 439 | *Maniola halicarnassus* | yes | x |  |  | x |  |  |  |  |
| 440 | *Erebia edda* | no |  |  |  | x |  |  |  |  |
| 441 | *Erebia fasciata* | no |  |  | x | x |  |  |  |  |
| 442 | *Erebia discoidalis* | no |  |  | x | x |  |  |  |  |
| 443 | *Erebia rossii* | no |  | x | x | x |  |  |  |  |
| 444 | *Erebia cyclopia* | no |  | x |  | x |  |  |  |  |
| 445 | *Erebia embla* | no |  | x |  | x |  |  |  |  |
| 446 | *Erebia disa* | no | x | x | x | x |  |  |  |  |
| 447 | *Erebia meolans* | no | x | x | x | x |  |  |  |  |
| 448 | *Erebia dabanensis* | no |  | x |  | x |  |  |  |  |
| 449 | *Erebia jeniseiensis* | no |  | x |  |  |  |  |  |  |
| 450 | *Erebia claudina* | no | x | x | x | x |  |  |  |  |
| 451 | *Erebia manto* | no | x | x | x | x |  |  |  |  |
| 452 | *Erebia ottomana* | no | x | x | x | x |  |  |  |  |
| 453 | *Erebia hispania* | no | x | x | x | x |  |  |  |  |
| 454 | *Erebia rondoui* | no | x | x | x | x |  |  |  |  |
| 455 | *Erebia callias* | no | x | x | x | x |  |  |  |  |
| 456 | *Erebia tyndarus* | yes | x | x | x | x | x | x | x | x |
| 457 | *Erebia cassioides* | yes | x | x | x | x | x | x | x | x |
| 458 | *Erebia nivalis* | no | x | x | x | x |  |  |  |  |
| 459 | *Erebia neleus* | no |  |  |  | x |  |  |  |  |
| 460 | *Erebia calcaria* | no | x | x | x | x |  |  |  |  |
| 461 | *Erebia arvernensis* | no | x | x | x | x |  |  |  |  |
| 462 | *Erebia oeme* | no | x | x | x | x |  |  |  |  |
| 463 | *Erebia gorge* | no | x | x | x | x |  |  |  |  |
| 464 | *Erebia sthennyo* | no | x | x | x | x |  |  |  |  |
| 465 | *Erebia pandrose* | no | x | x | x | x |  |  |  |  |
| 466 | *Erebia eriphyle* | no | x | x | x | x |  |  |  |  |
| 467 | *Erebia epistygne* | no | x | x | x | x |  |  |  |  |
| 468 | *Erebia euryale* | no | x | x | x | x |  |  |  |  |
| 469 | *Erebia palarica* | no | x | x | x | x |  |  |  |  |
| 470 | *Erebia ligea* | no | x | x | x | x |  |  |  |  |
| 471 | *Erebia pluto* | no | x | x | x | x |  |  |  |  |
| 472 | *Erebia aethiopellus* | no | x | x | x | x |  |  |  |  |
| 473 | *Erebia gorgone* | no |  | x |  | x |  |  |  |  |
| 474 | *Erebia rhodopensis* | no | x | x | x | x |  |  |  |  |
| 475 | *Erebia mnestra* | no | x | x | x | x |  |  |  |  |
| 476 | *Erebia alberganus* | no | x | x | x | x |  |  |  |  |
| 477 | *Erebia sudetica* | no | x | x | x | x |  |  |  |  |
| 478 | *Erebia melampus* | no | x | x | x | x |  |  |  |  |
| 479 | *Erebia triarius* | no | x | x | x | x |  |  |  |  |
| 480 | *Erebia polaris* | no |  | x |  | x |  |  |  |  |
| 481 | *Erebia medusa* | no | x | x | x | x |  |  |  |  |
| 482 | *Erebia aethiops* | no | x | x | x | x |  |  |  |  |
| 483 | *Erebia pharte* | no | x | x | x | x |  |  |  |  |
| 484 | *Erebia christi* | no | x | x | x | x |  |  |  |  |
| 485 | *Erebia orientalis* | no | x | x | x | x |  |  |  |  |
| 486 | *Erebia epiphron* | no | x | x | x | x |  |  |  |  |
| 487 | *Erebia flavofasciata* | no | x | x | x | x |  |  |  |  |
| 488 | *Erebia montanus* | no | x | x | x | x |  |  |  |  |
| 489 | *Erebia styx* | no | x | x | x | x |  |  |  |  |
| 490 | *Erebia stirius* | no | x | x | x | x |  |  |  |  |
| 491 | *Erebia scipio* | no | x | x | x | x |  |  |  |  |
| 492 | *Erebia pronoe* | no | x | x | x | x |  |  |  |  |
| 493 | *Erebia melas* | no | x | x | x | x |  |  |  |  |
| 494 | *Erebia lefebvrei* | no | x | x | x | x |  |  |  |  |
| 495 | *Erebia zapateri* | no | x | x | x | x |  |  |  |  |
| 496 | *Erebia neoridas* | no | x | x | x | x |  |  |  |  |
