# Supplementary material for: Assessing the aesthetic attractivity of European butterflies: A web-based survey protocol
Source: PLoS One. 2023 May 11;18(5):e0283360. doi: 10.1371/journal.pone.0283360 (PMC10174575; doi:10.1371/journal.pone.0283360)
Supplement: S1 Appendix — All the questions proposed to the participants during the test are collected together with their answer options in this file. (DOCX) [file pone.0283360.s002.docx]

**S1 Appendix**

In this appendix all the questions proposed to the participants during the test (available on the website <https://www.unveiling.eu/>) are collected together with their answer options.

Section n.1 : **Personal data**

- You’re…? male / female / other
- How old are you? 1- 99
- And your nationality is…? Italian / British / German / French / Spanish / Other
- What's your qualification? Compulsory education / graduation / bachelor’s or master's degree / PhD
- Which of the following occupational fields appeals to you the most? Services-administration / productive (crafts,trade,etc.) / scientific technical / teacher / artistic-cultural / student / other
- What number do you see? 71 / 74

Section n.2 : **Ranking**

In this section participants are presented with a panel of 9 pictures of different butterfly species, and they are asked to attribute a score (from 1 to 10) to each of them in response to the question “How beautiful do they look to you?”. The list of all images of the different species is available in Appendix 1

Section n.3 : **Single morphological features**

In this section, 10 pairs of butterfly-drawings created *ad hoc* are shown to each participant in random positions (left-right). The list of all drawings submitted is available in Appendix 2

Section n.4 : **Emotional engagement**

emotion that comes closest to what they feel, also quantifying the emotional intensity in a scale range from 1 to 10. The possibilities of response are: joy / feeling of change / fear / disgust / cuteness / confusion / awe / none of these.

Every 2,500 answers, the pictures will be changed to obtain a higher representativity of the butterfly species diversity.

Section n.5 **: Dispositional variables. Interests and inclinations**

A questionnaire in which a 5-point Likert scale (strongly agree; agree; neither agree nor disagree; disagree; strongly disagree) from which to select a response is presented to the participants. Participants have to agree/disagree with 18 statements:

1. I find apps with scientific content (e.g. apps to identify constellations or to spot animal or plant species) uninteresting!
2. When I go on nature walks, I love to take pictures of the species of animals and plants I encounter.
3. I do not believe that my expertise in scientific matters is superior to that of the average person.
4. I think I have a deeper knowledge of the butterfly world than the average person.
5. I don't find it particularly interesting to observe animals in their natural habitat.
6. If I were to define myself, I'd say I have an interest in science.
7. I don't think I could stare at a beautiful painting too long: it would bore me, after a while!
8. Practicing some form of art (painting, acting, playing, sculpting, etc.) is not among my favorite activities.
9. Being in a natural environment (instead of a city, for example) gives me a sense of wellness.
10. I really enjoy creating beautiful things in one or more artistic fields such as visual art, music, dance, writing, etc.
11. In my spare time, I often go visiting art galleries or museums (not counting the recent COVID-19 restrictions).
12. I regularly read books or art magazines (also online).
13. I think my moods are very much influenced by the beauty of my surroundings.
14. I really value the scientific point of view in my everyday life.
15. I don't think I'm interested in art and beauty more than the average.
16. If I had to give a definition of myself, I would say that I am interested in art.
17. When I see something beautiful, in my everyday life, I rarely get passionate about it.
18. When I come across a programme on television or online about science, I rarely get excited.
