# Supplementary material for: Assessing the aesthetic attractivity of European butterflies: A web-based survey protocol
Source: PLoS One. 2023 May 11;18(5):e0283360. doi: 10.1371/journal.pone.0283360 (PMC10174575; doi:10.1371/journal.pone.0283360)
Supplement: S2 Appendix — All the couples of drawings with respective features analysis are available in this file. (DOCX) [file pone.0283360.s003.docx]

**S3 Appendix**

In this appendix all pairs of butterfly drawings with natural and altered appearance are listed and subdivided into paragraphs, where it is made explicit which morphological feature of the species has been altered and how. Drawings are presented according to Table 1 order.

**Table 1. Single morphological features analysed in the third section of the “Unveiling” test.**

| **Morphological features** | **Butterfly species** | **Number of cases** |
| --- | --- | --- |
| Butterfly dimension | *Nymphalis antiopa* | 2 (natural, 36% smaller) |
| Colours of the wings contrast intensity | *Charaxes jasius* | 3 (natural, 40% brighter and 0% contrast, 40% less bright and 40% more contrast) |
| Grouping and order of the design patterns of the wings | *Charaxes jasius; Erebia medusa* | 2 (natural, modified with an unordered and random arrangement of wing elements) |
| Forewing / hindwing proportion | *Kirinia roxelana* | 2 (natural, modified with altered fore wing / hind wing proportion) |
| Presence or absence of wings eyespots | *Aglais io; Erebia medusa* | 2 (natural, modified aspect without wing eyespots) |
| Wings eyespots dimensions | *Aglais io; Erebia medusa* | 3; 2 (natural, modified aspect with 100% smaller (only for *A. io*) eyespots, modified aspect with 100% bigger eyespots) |
| Presence or absence of wings tails | *Iphiclides podalirius; Charaxes jasius* | 2 (natural, modified aspect without wing tails) |
| Wings tails length | *Iphiclides podalirius; Charaxes jasius* | 2 (natural, modified with 20% longer wing tails for *I. podalirius* and 15% longer wing tails for *C. jasius*) |
| Smooth or jagged wings edges | *Polygonia c-album* | 3 (natural, modified with jagged edges, modified with smooth edges) |

Single features analysed in the third section of the “Unveiling” test, butterfly species associated with them and number of cases for each feature.

**Morphological feature:** butterfly dimension

**Butterfly species:** *Nymphalis antiopa*

(A) *
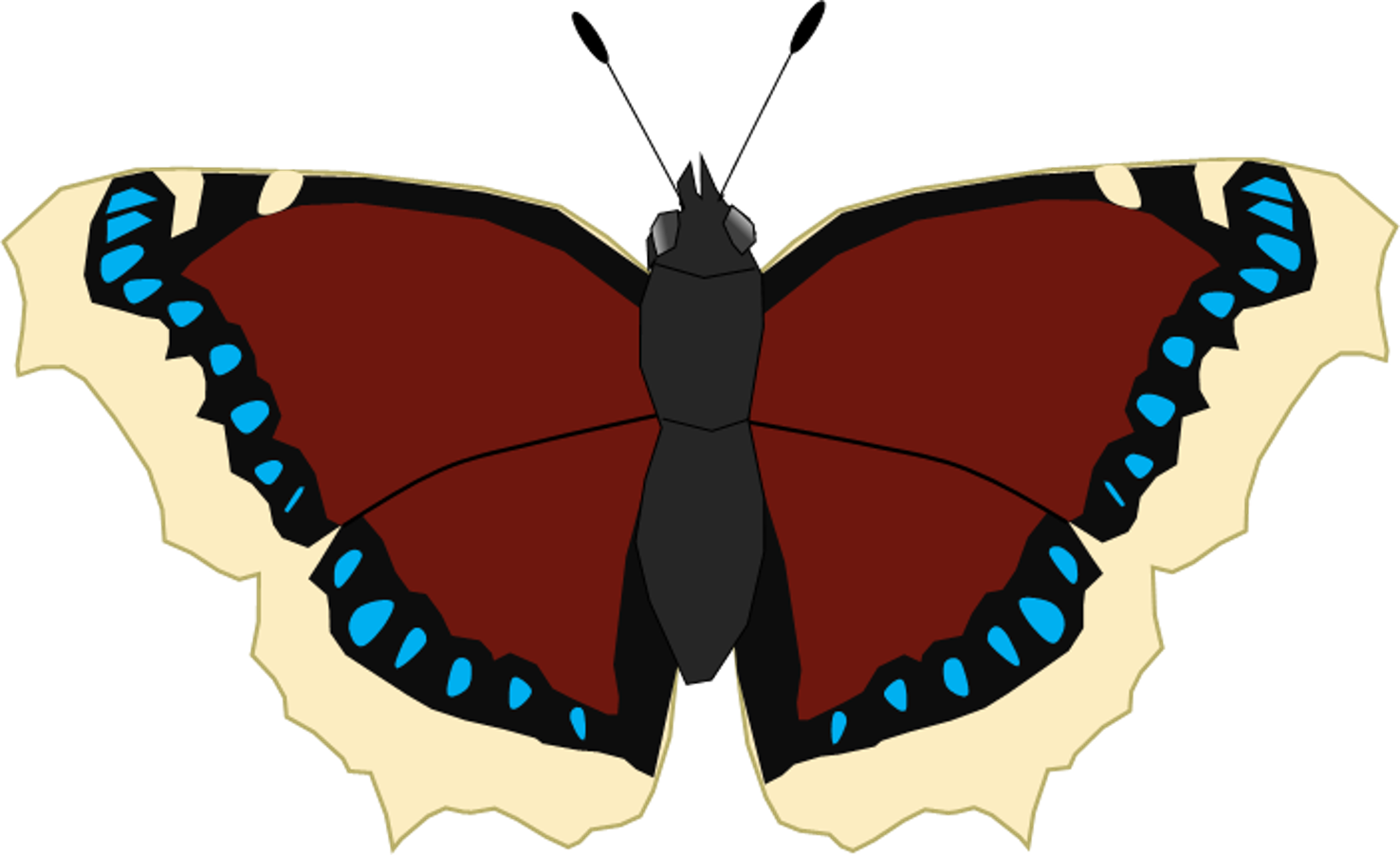
* (B)*
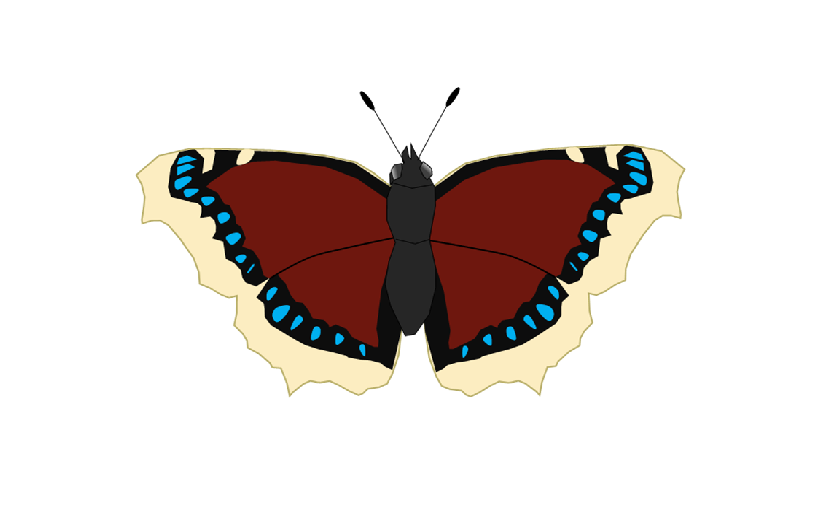
*

**Fig 1. Two *Nymphalis antiopa* drawings.** Two *Nymphalis antiopa* drawings with different dimensions (A) standard drawing dimension (B) modified drawing dimensions: butterfly B is 36% smaller than the other butterfly drawing.

**Morphological feature:** colours of the wings contrast intensity

**Butterfly species:** *Charaxes jasius*

(A)*
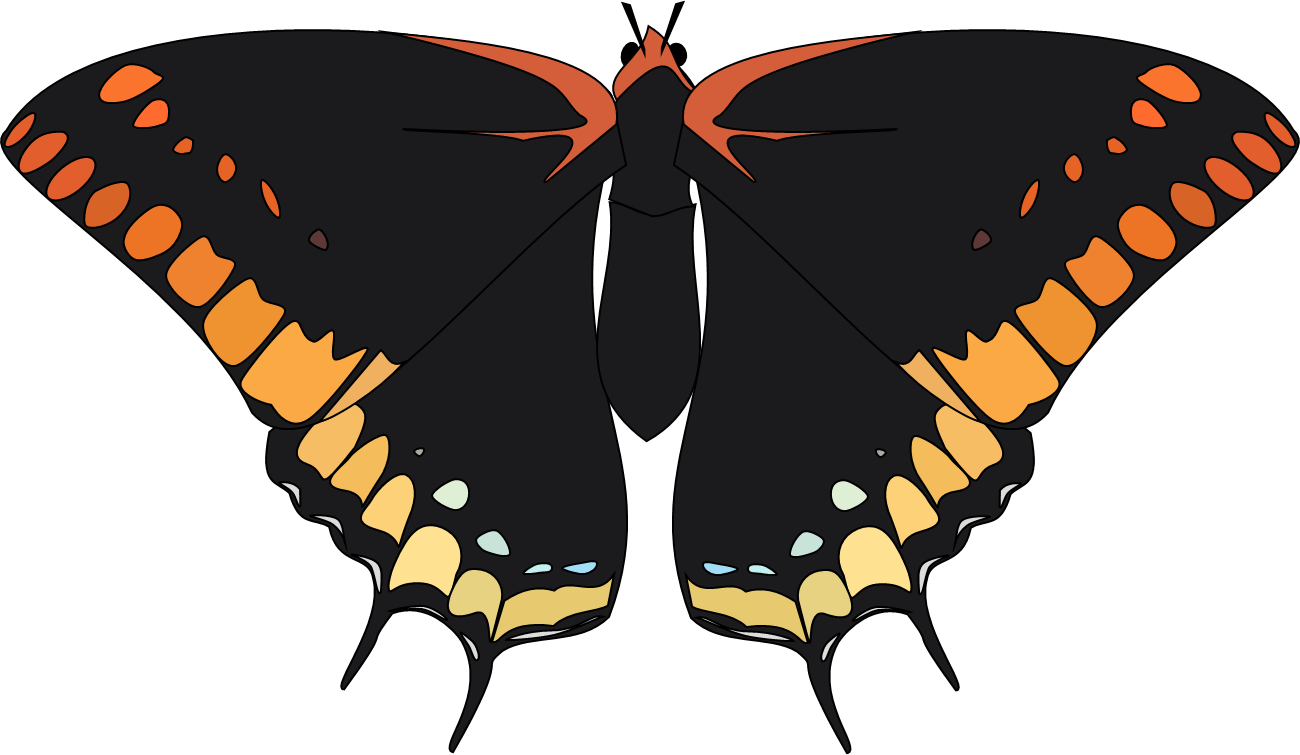
*

(B) *
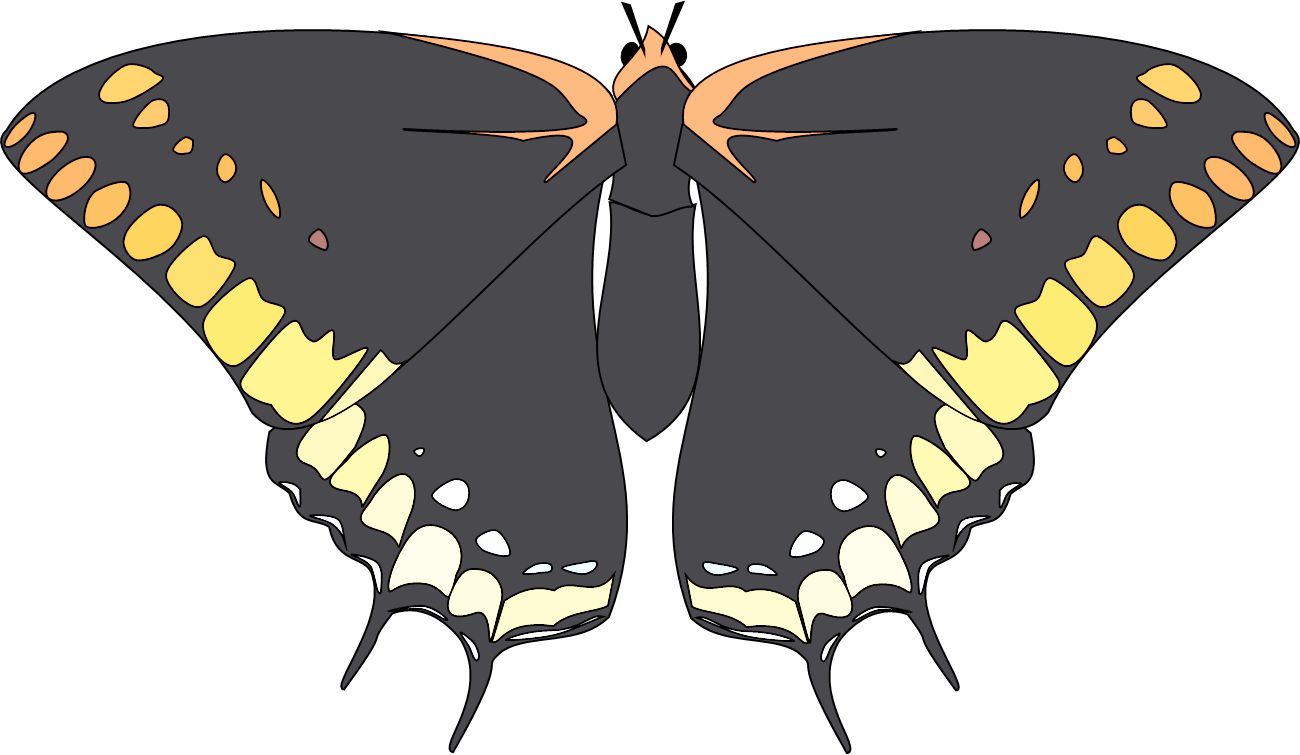
*(C)*
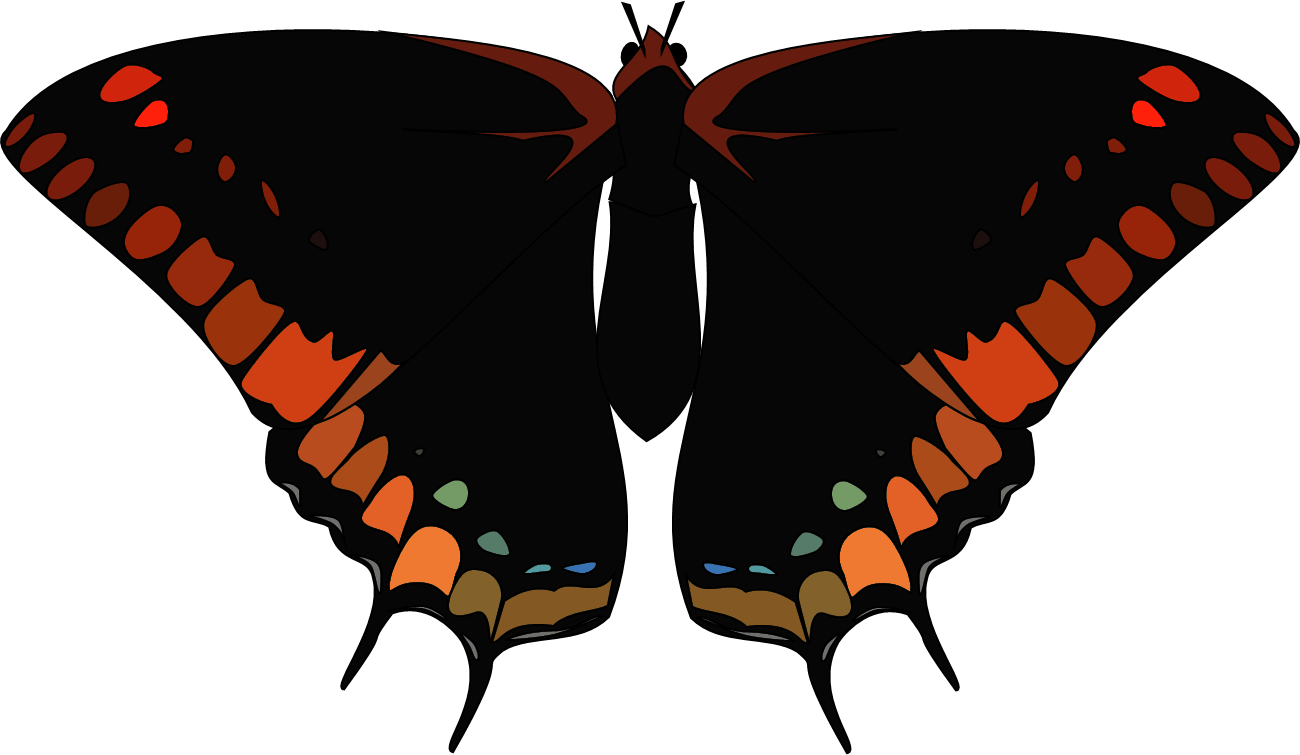
*

**Fig 2. Three *Charaxes jasius* drawings.** Three *Charaxes jasius* drawings with different colours of the wings contrast intensity (A) natural aspect (B) modified colour contrast (40% brighter and 0% contrast) (C) modified colour contrast (40% less bright and 40% more contrast).

**Morphological feature:** grouping and order of the design patterns of the wings

**Butterfly species:** *Charaxes jasius*

(A)*
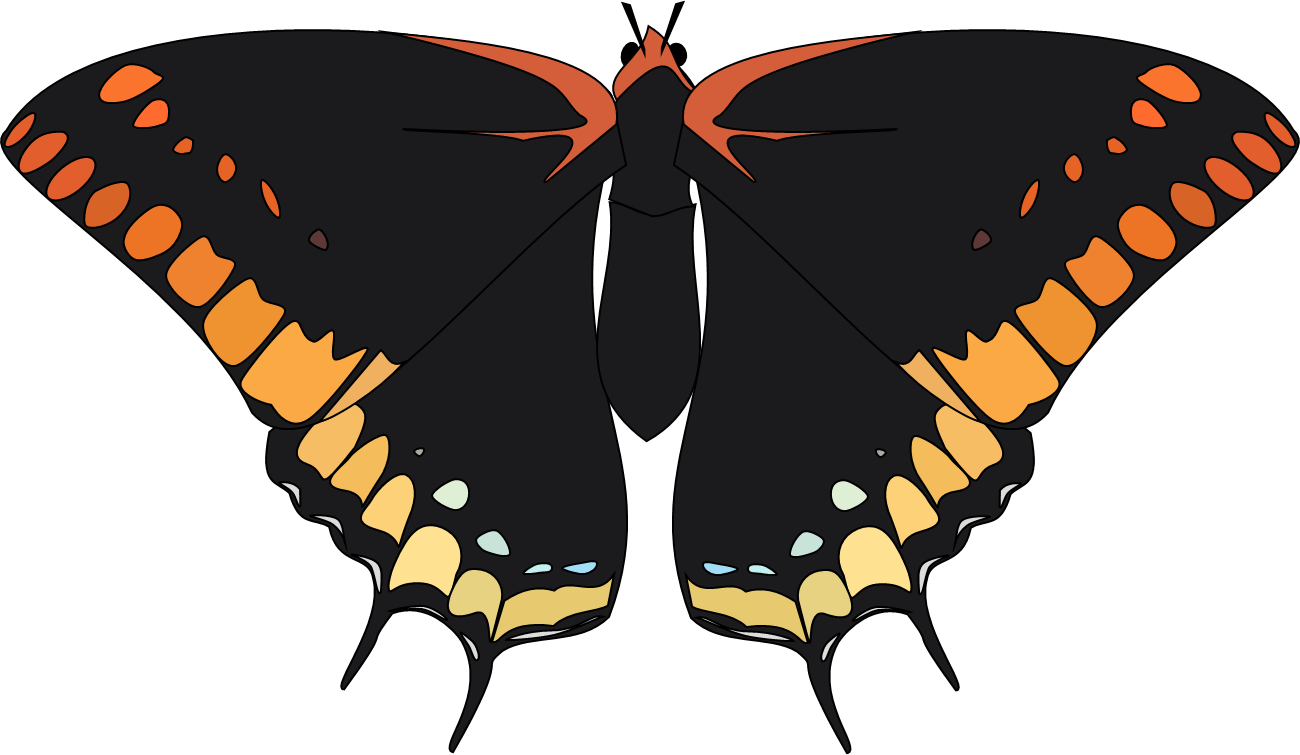
*(B) *
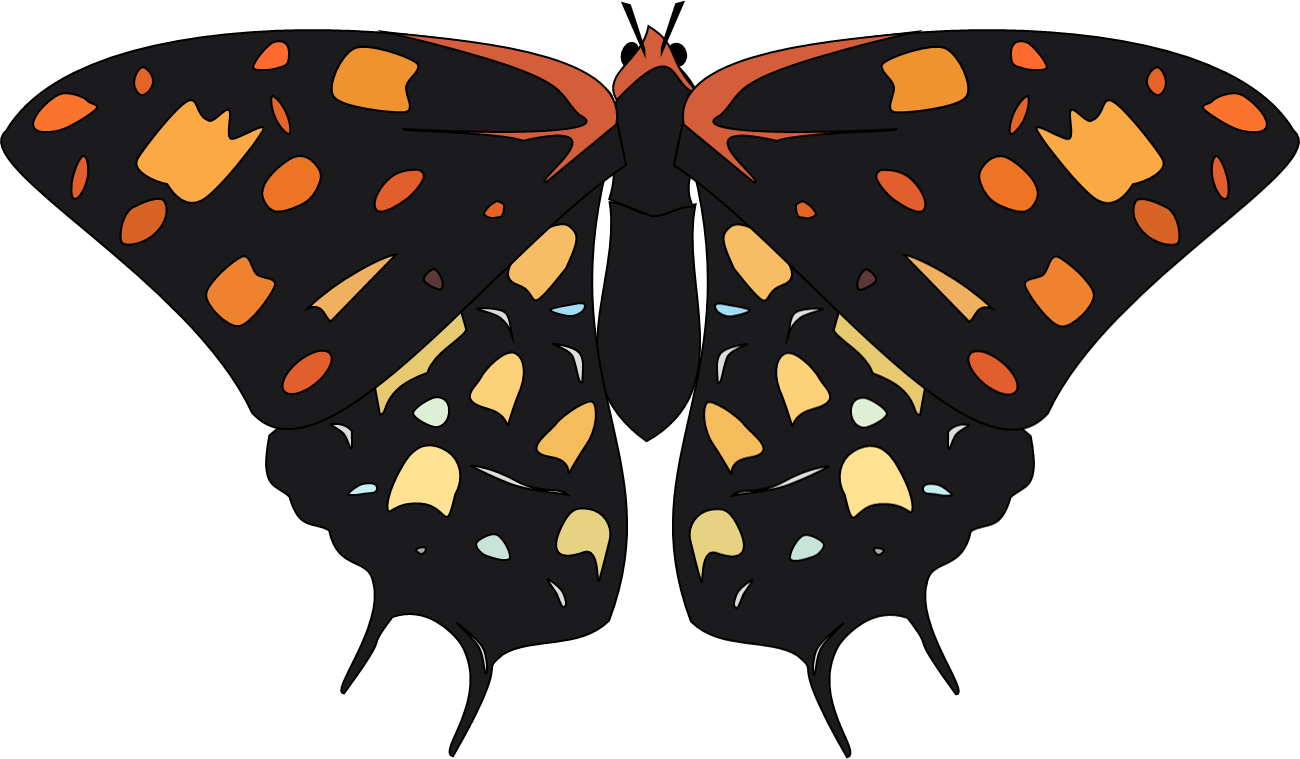
*

**Fig 3. Two *Charaxes jasius* drawings.** Two *Charaxes jasius* drawings with different Grouping and order of the design patterns of the wings (A) natural aspect (B) modified with an unordered and random arrangement of wing elements.

**Butterfly species:** *Erebia medusa*

(A)*
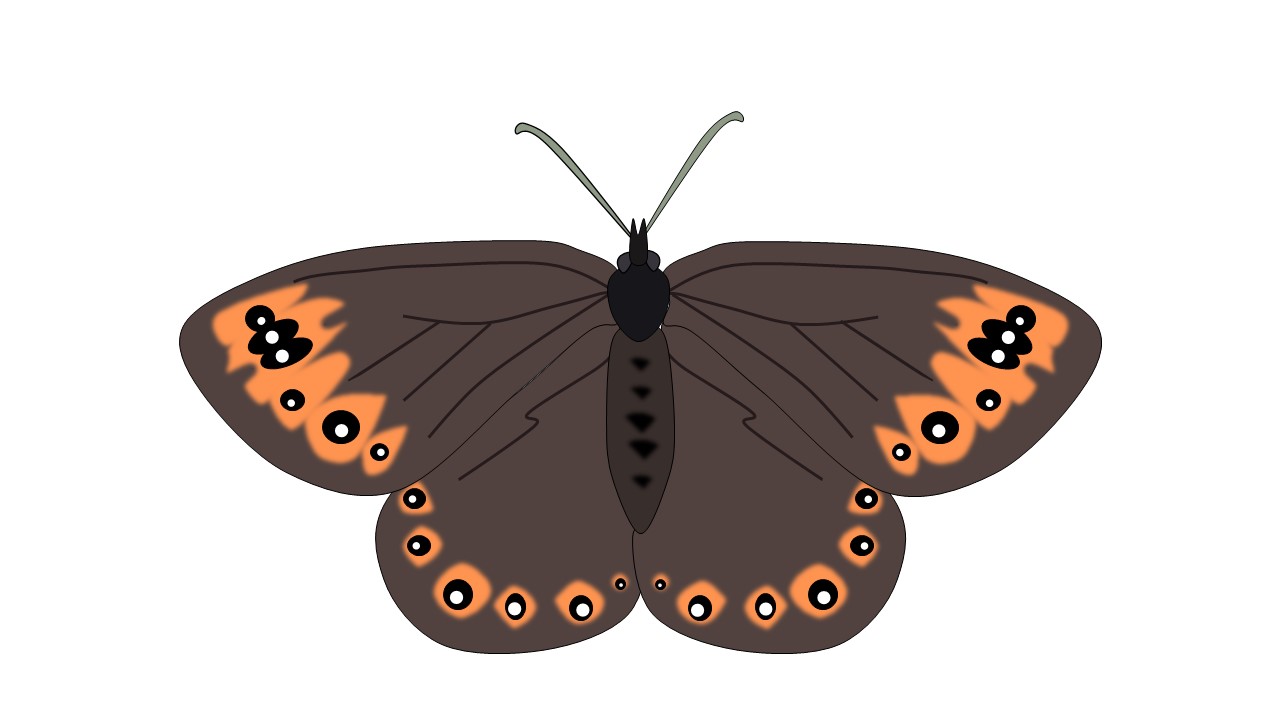
*(B)*
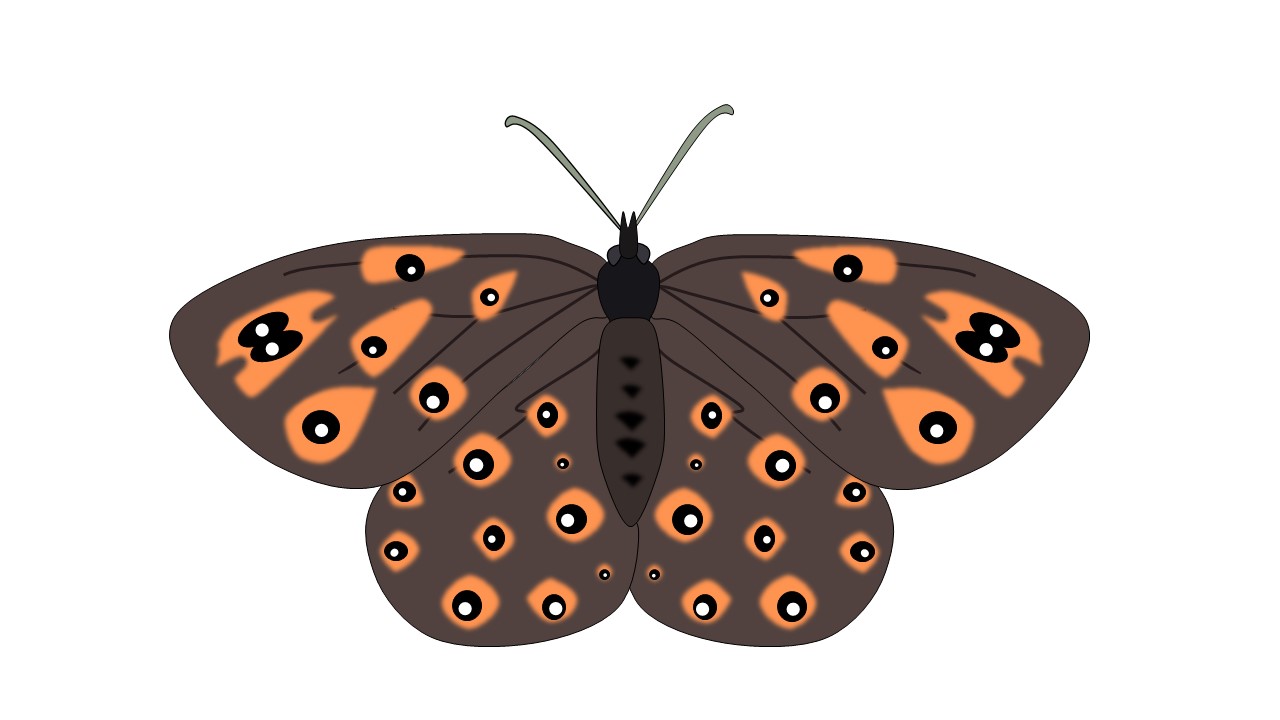
*

**Fig 4. Two *Erebia medusa* drawings.** Two *Erebia medusa* drawings with different grouping and order of the design patterns of the wings (A) natural aspect (B) modified with an unordered and random arrangement of wing eyespots (butterfly B has 10 more eyespots than butterfly A).

**Morphological feature:** fore wing / hind wing proportion

**Butterfly species:** *Kirinia roxelana*

(A)*
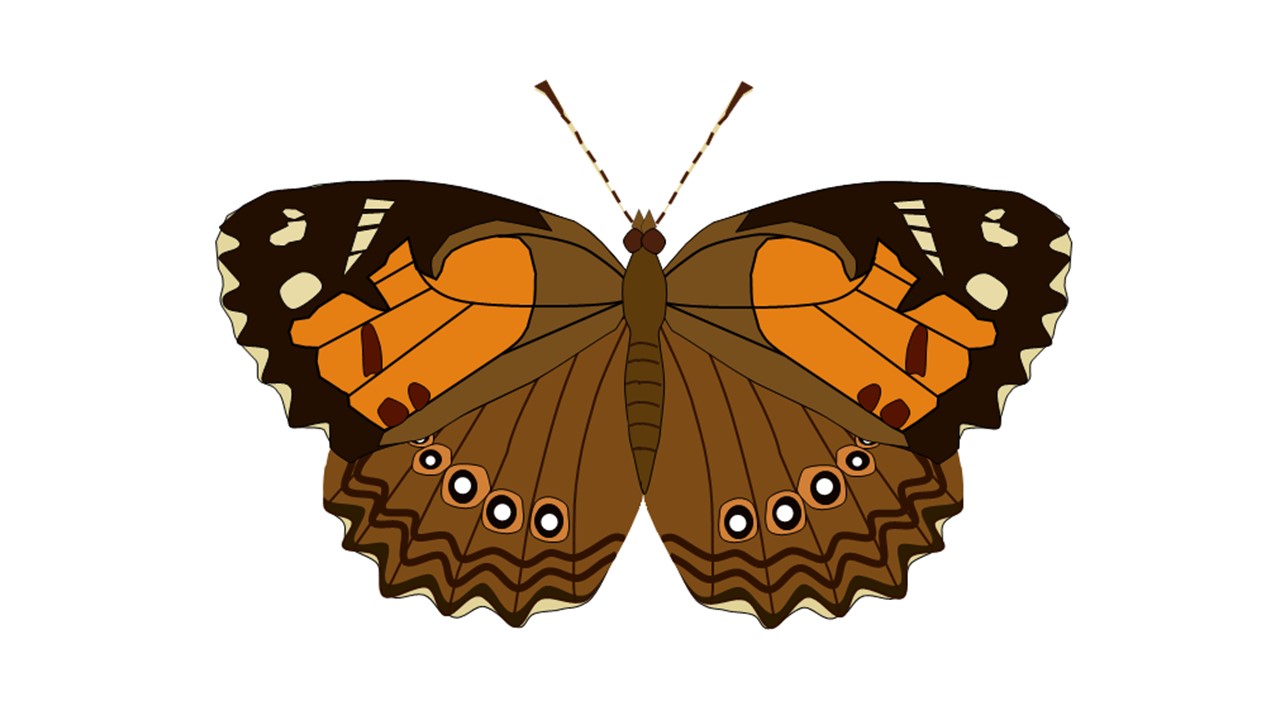
*(B)*
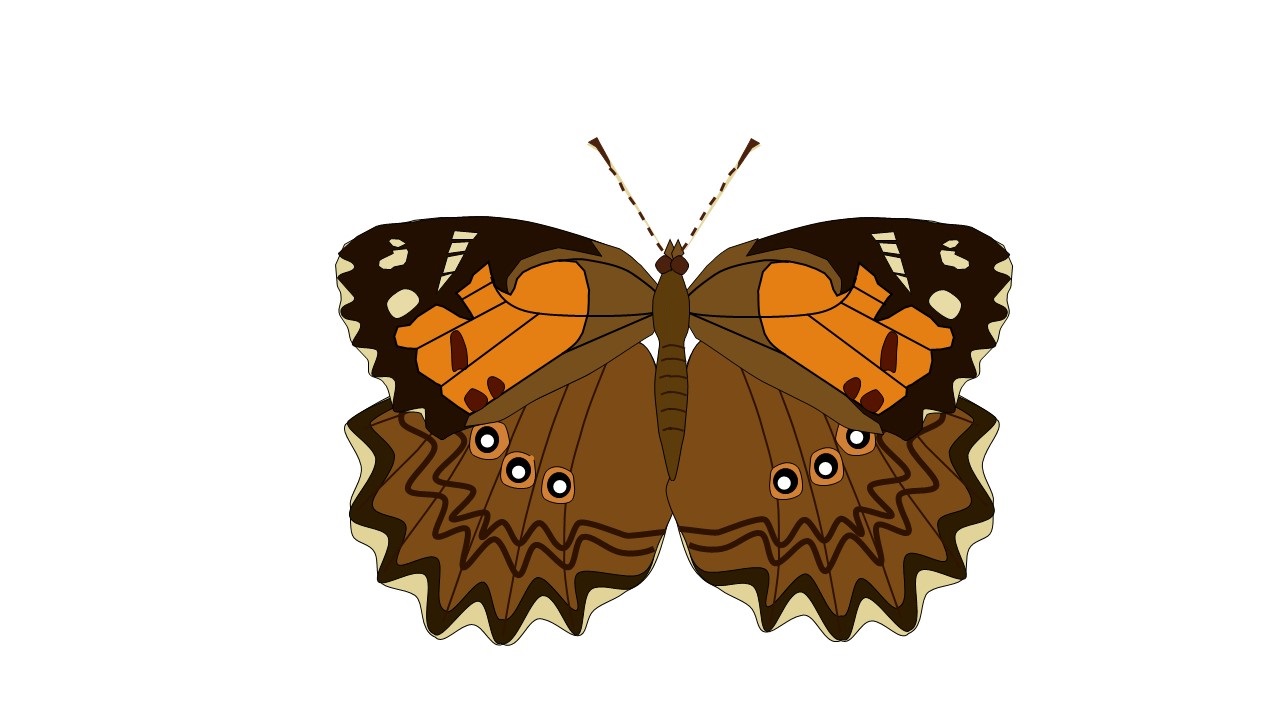
*

**Fig 5. Two** ***Kirinia roxelan*a drawings.** Two *Kirinia roxelana* drawings with different fore wing / hind wing proportions (A) natural aspect (B) modified with altered fore wing / hind wing proportion: bigger hind wings (7.5%) than natural dimensions.

**Morphological feature:** presence or absence of wings eyespots

**Butterfly species:** *Aglais io*

(A)*
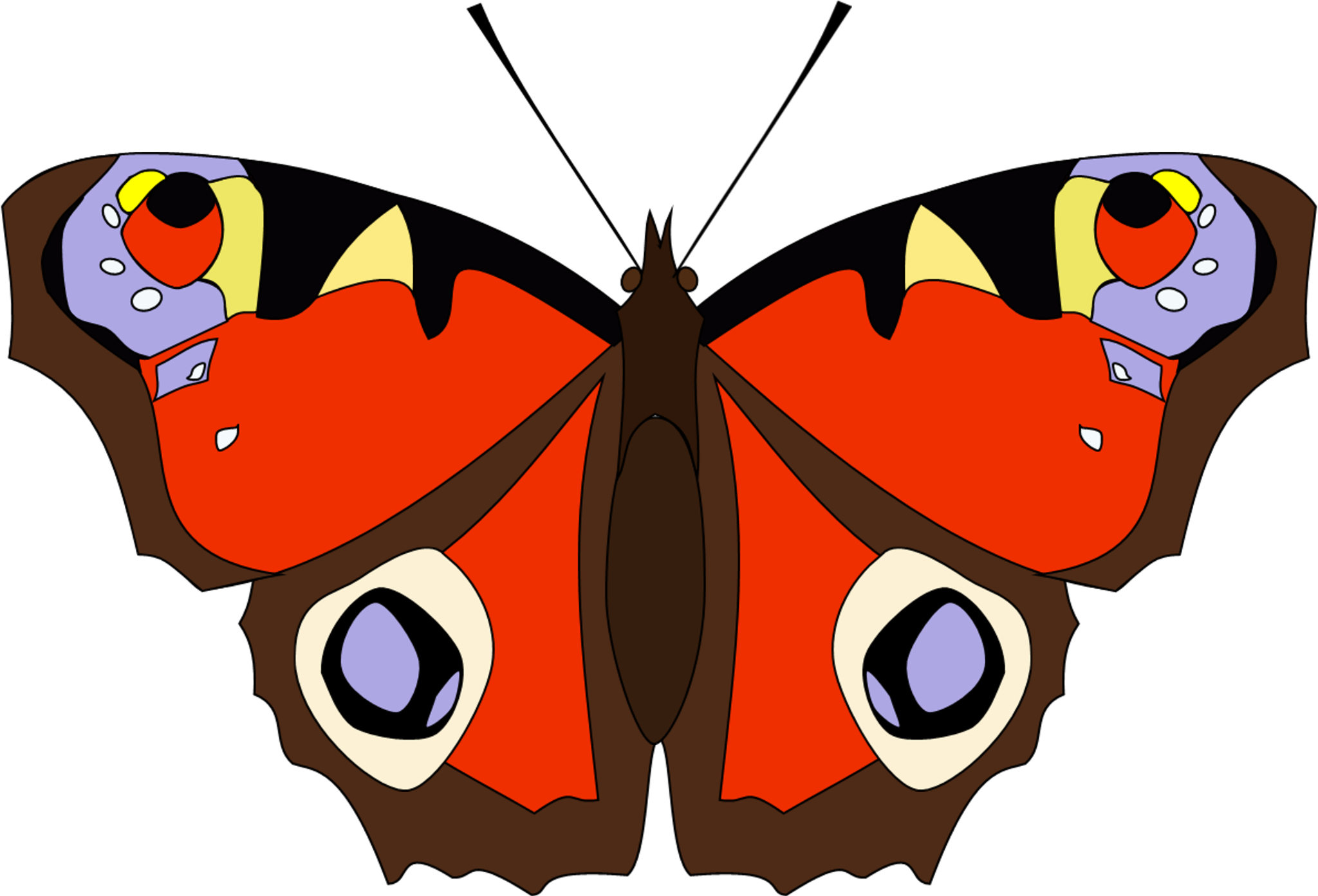
* (B)*
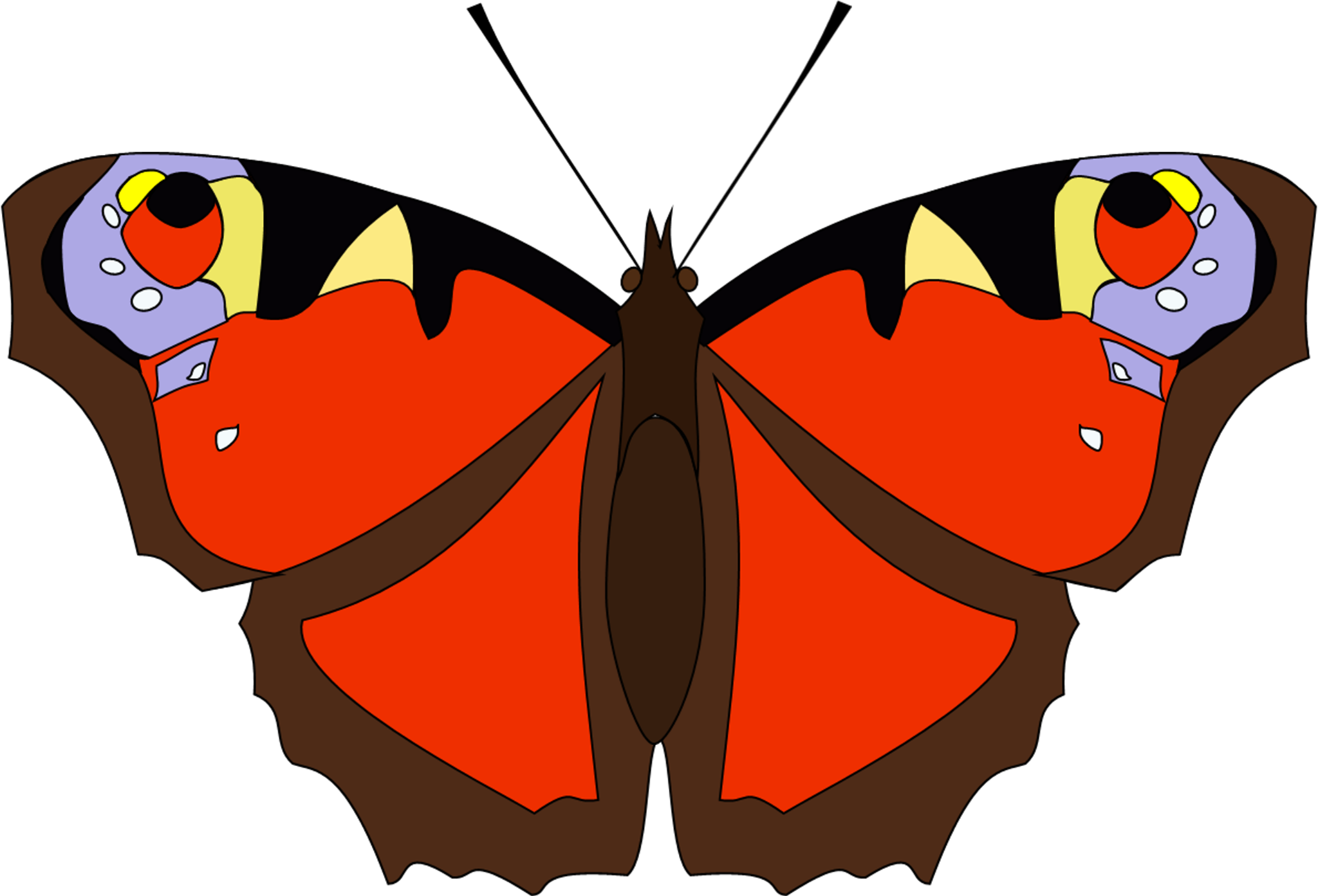
*

**Fig 6. Two *Aglais io* drawings.** Two *Aglais io* drawings with and without wing eyespots (A) natural aspect with wing eyespots (B) modified aspect without wing eyespots.

**Morphological feature:** presence or absence of wings eyespots

**Butterfly species:** *Erebia medusa*

(A)**
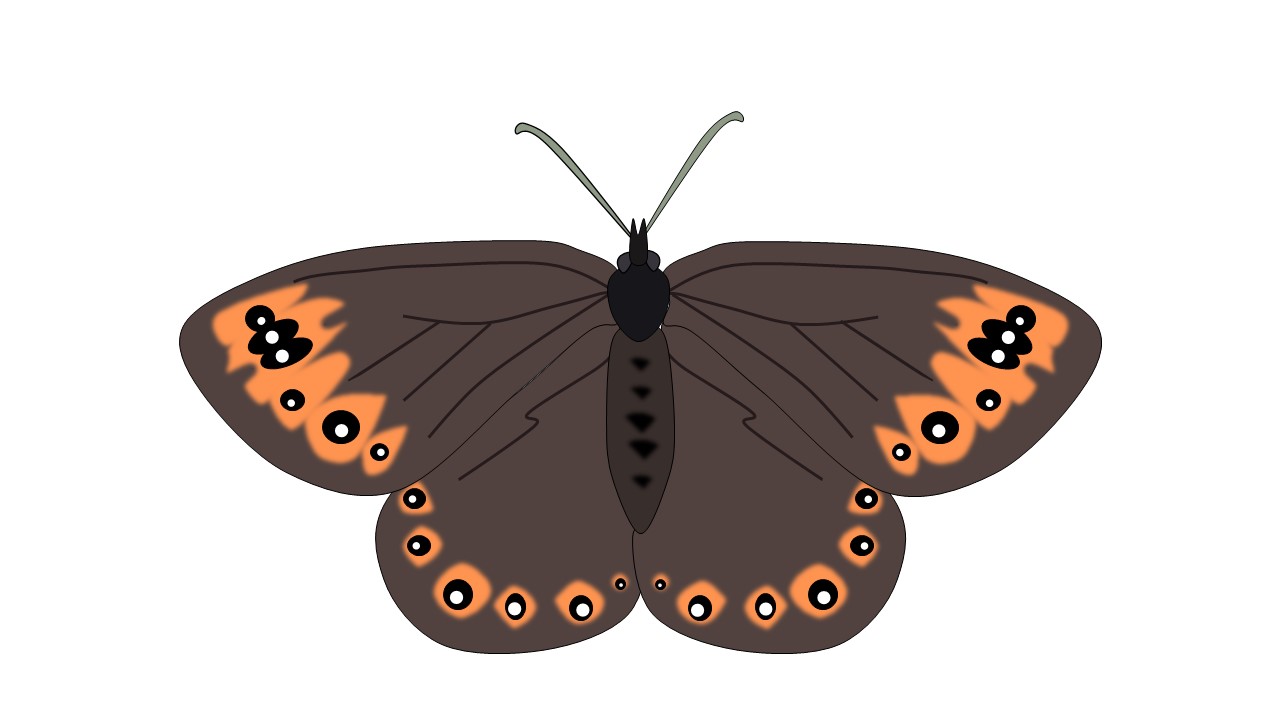
**(B)**
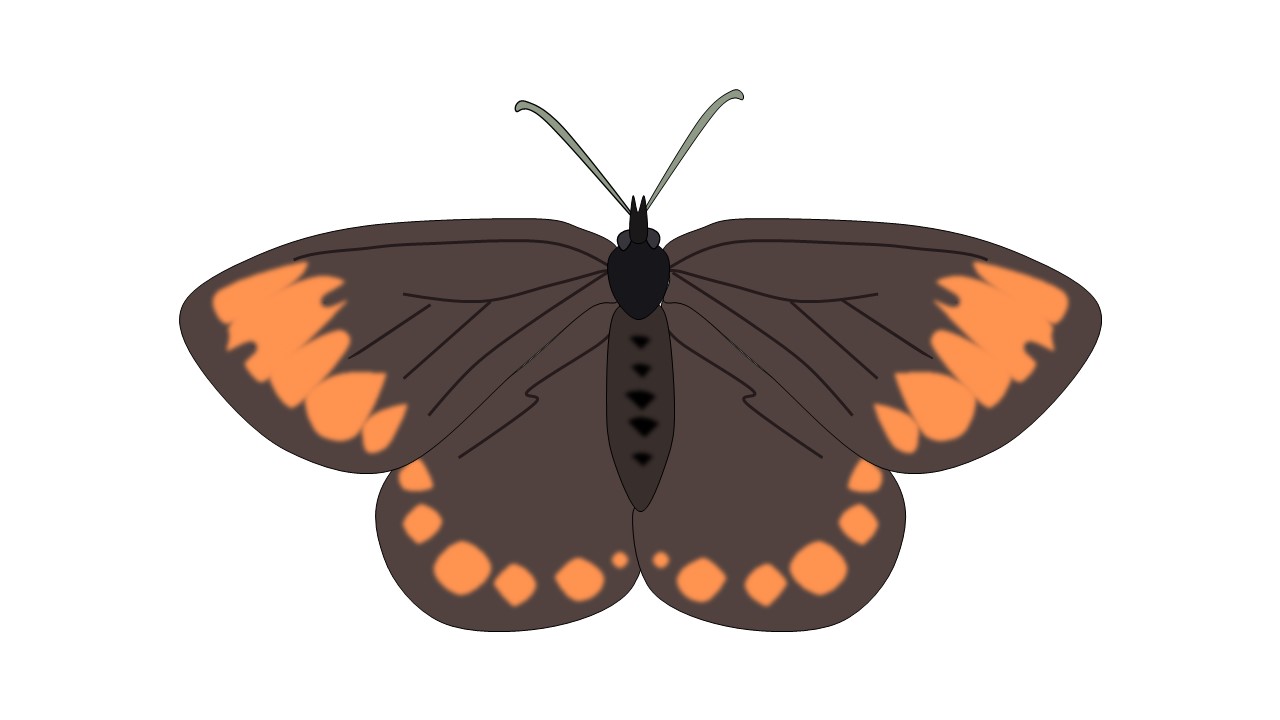
**

**Fig 7. Two *Erebia medusa* drawings.** Two *Erebia medusa* drawings with and without wing eyespots (A) natural aspect with wing eyespots (B) modified aspect without wing eyespots

**Morphological feature:** wings eyespots dimensions

**Butterfly species:** *Aglais io*

(A) *
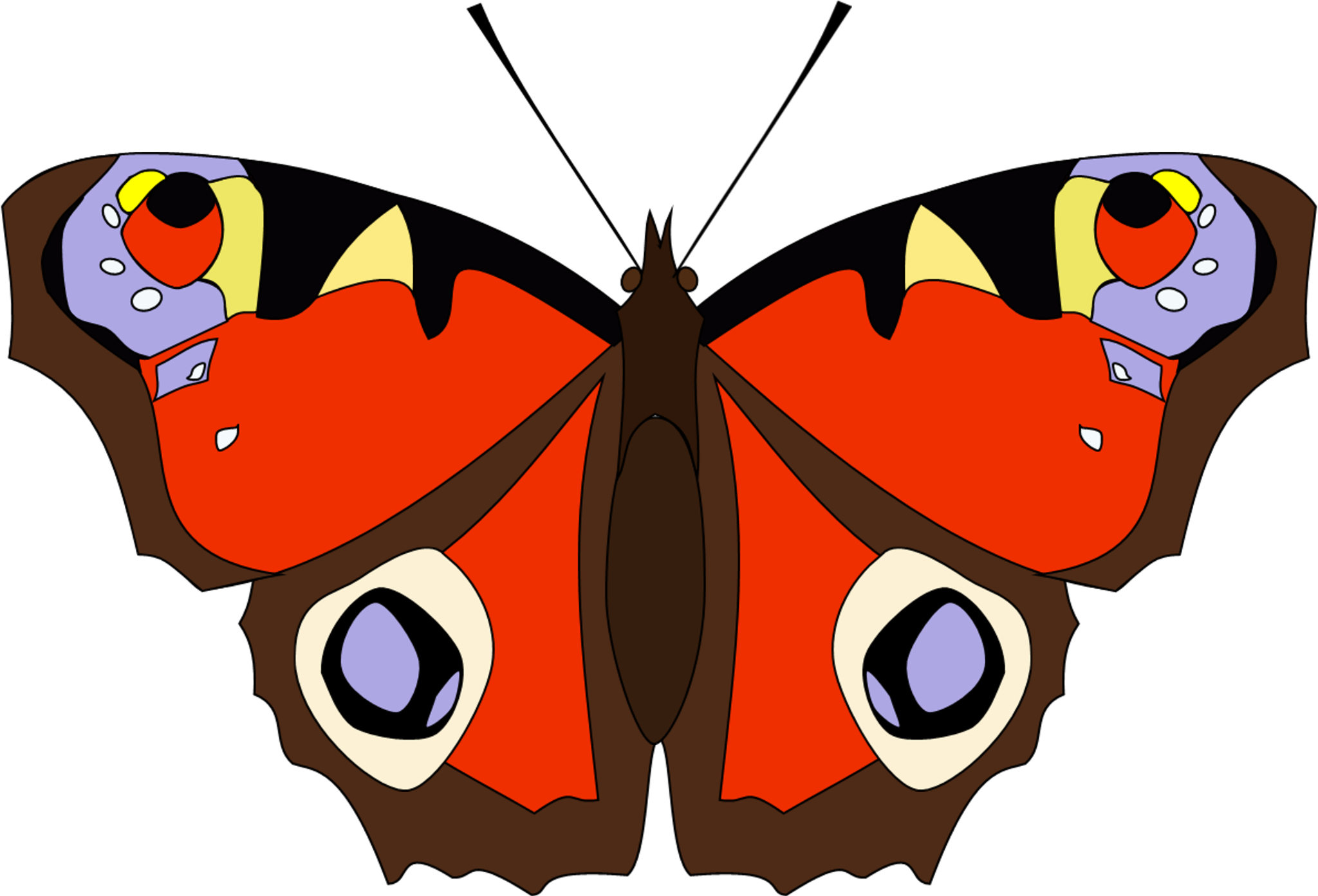
*

(B)
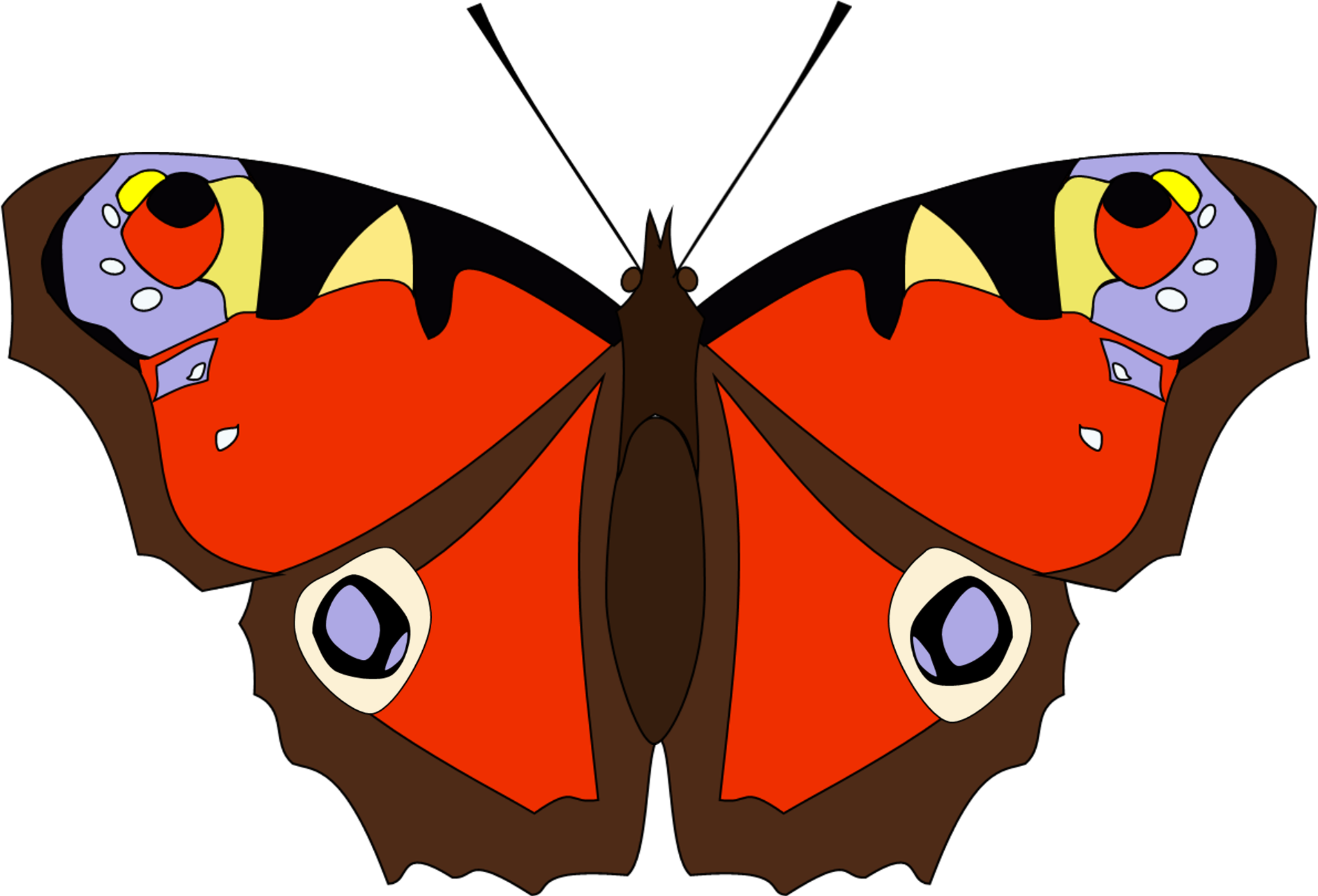
(C)*
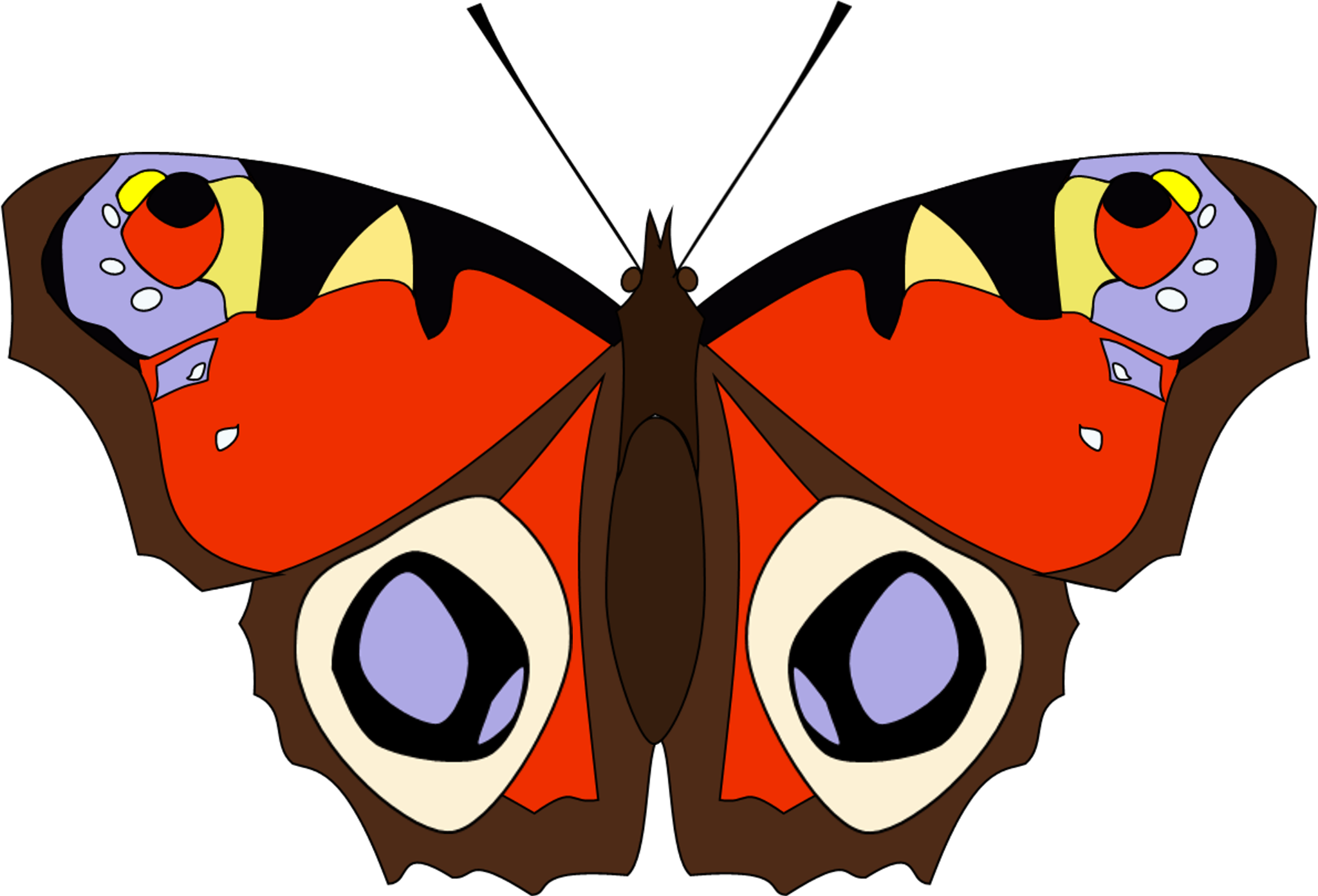
*

**Fig 8. Three *Aglais io* drawings.** Two *Aglais io* drawings with wings eyespots dimensions (A) natural aspect (B) modified aspect with 100% smaller eyespots (C) modified aspect with 100% bigger eyespots.

**Butterfly species:** *Erebia medusa*

(A)**
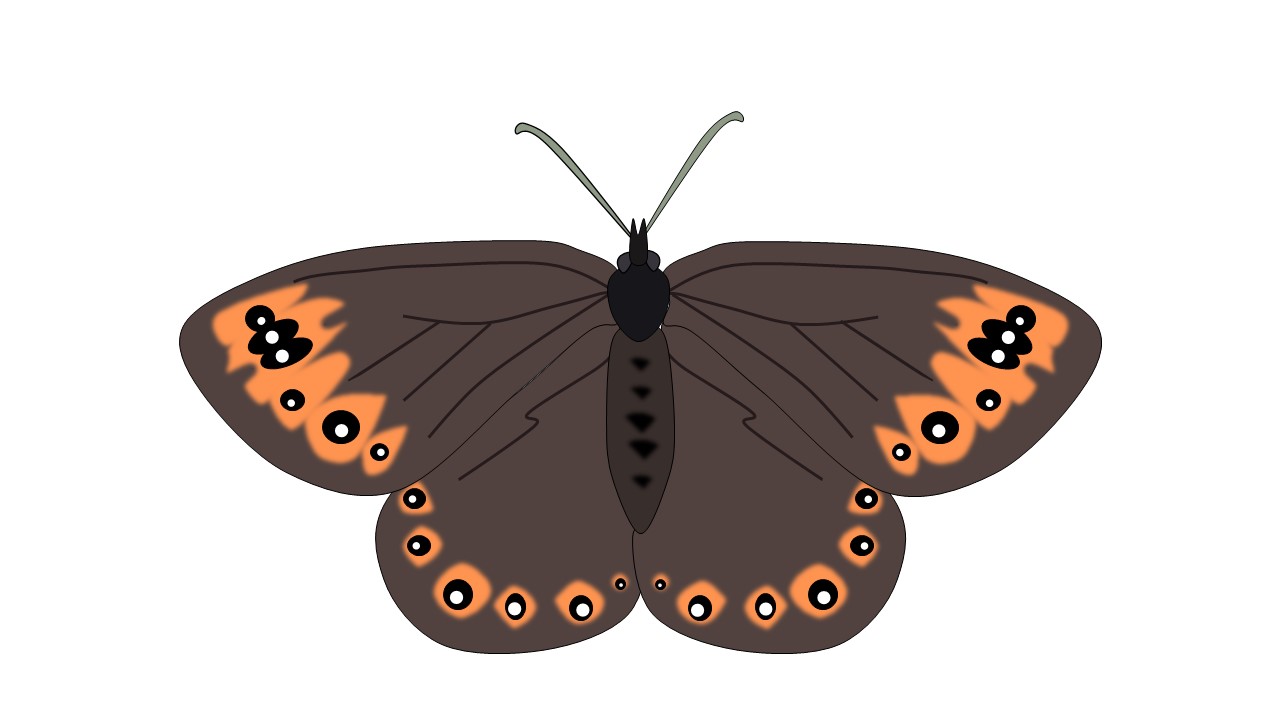
**(B)**
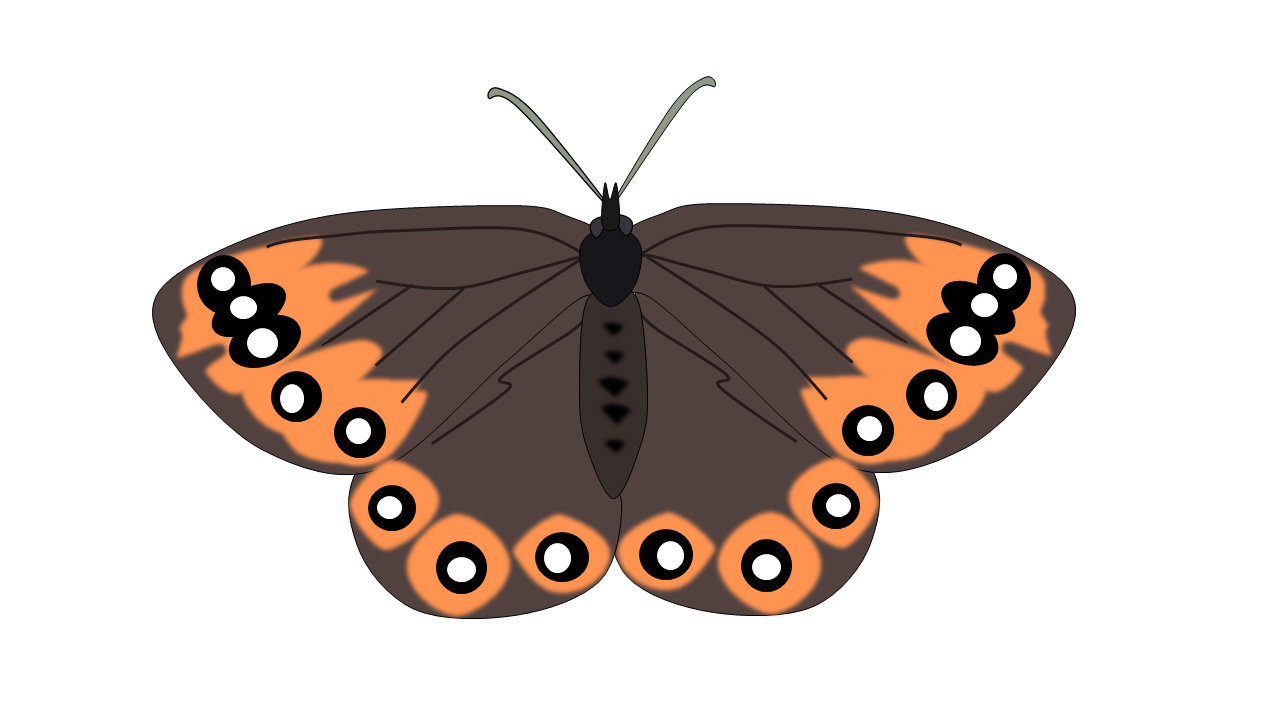
**

**Fig 9. Two *Erebia medusa* drawings.** Two *Erebia medusa* drawings with wing eyespots dimensions (A) natural aspect (B) modified with 100% bigger eyespots.

**Morphological feature:** presence or absence of wings tails

**Butterfly species:** *Iphiclides podalirius*

(A)*
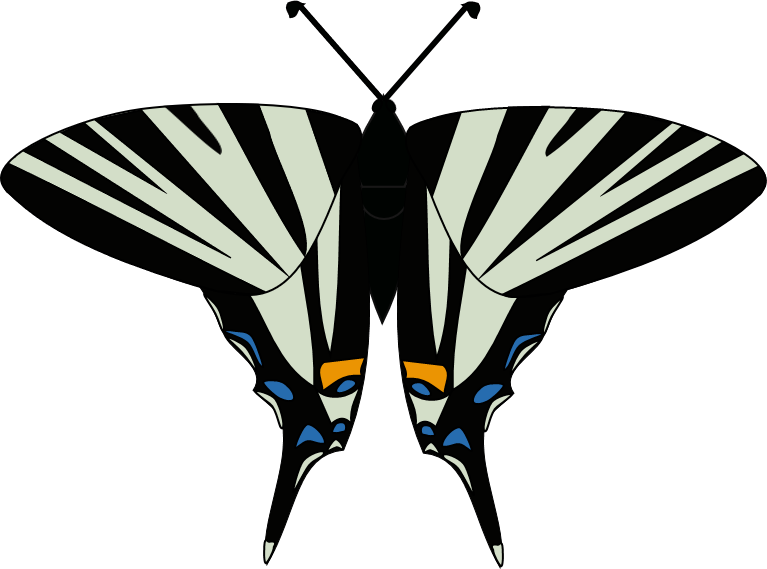
*  (B)*
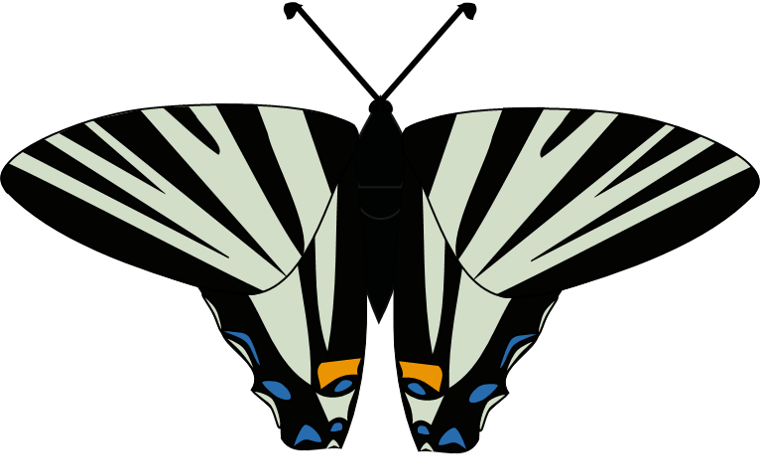
***Fig 10. Two *Iphiclides podalirius*** **drawings.** Two *Iphiclides podalirius* drawings with or without wing tails (A) natural aspect with wing tails (B) modified aspect without wing tails.

**Butterfly species:** *Charaxes jasius*

(A)*
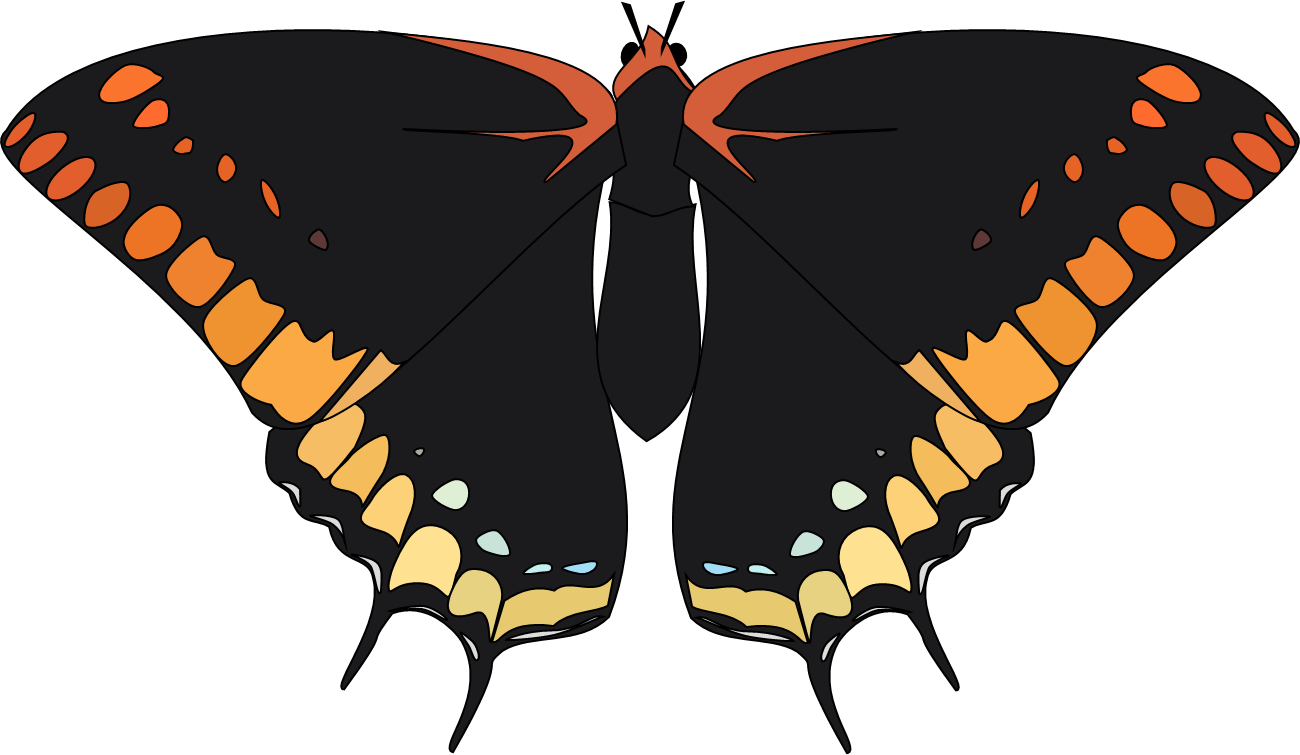
*  (B)*
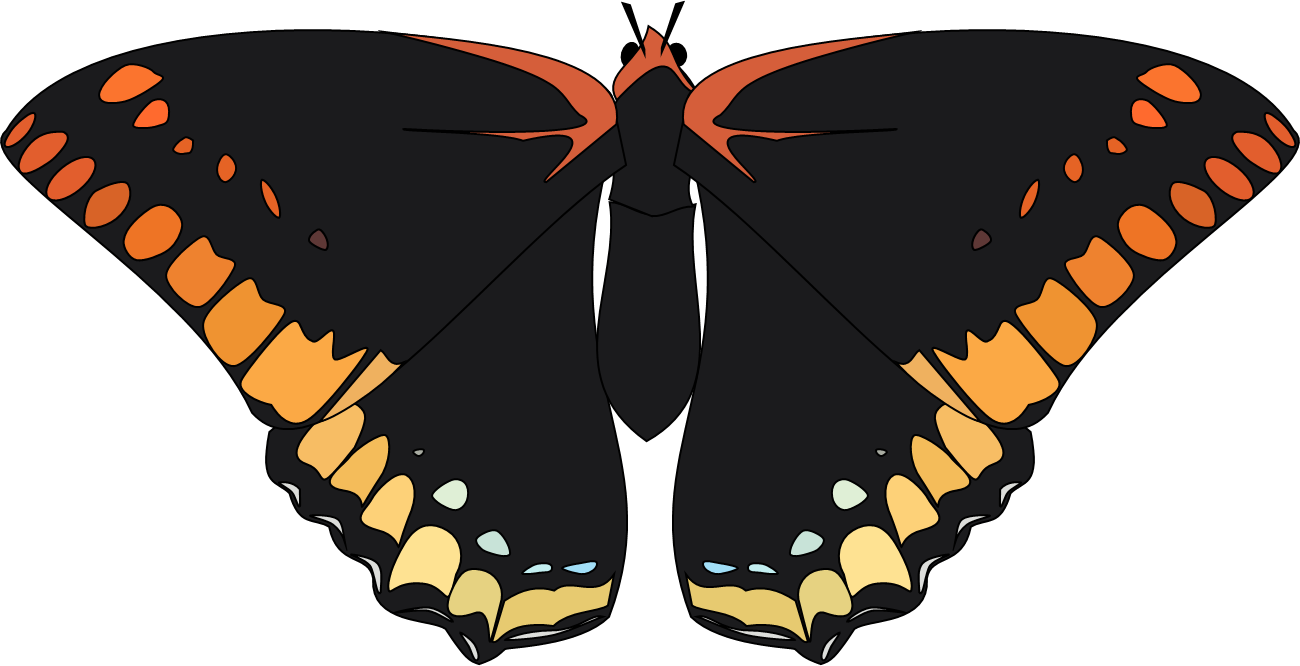
*

**Fig 11. Two *Charaxes jasius*** **drawings.** Two *Charaxes jasius* drawings with or without wing tails (A) natural aspect with wing tails (B) modified aspect without wing tails.

**Morphological feature:** wings tails length

**Butterfly species:** *Iphiclides podalirius*

(A)*
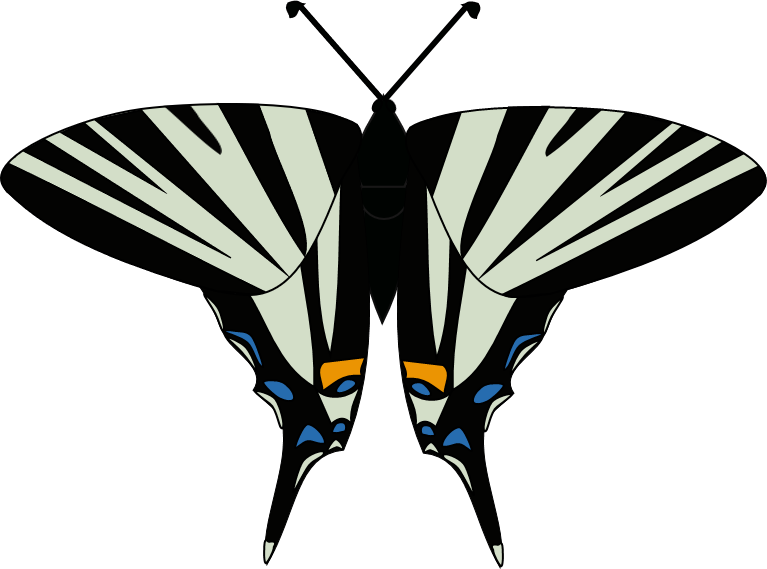
* (B) *
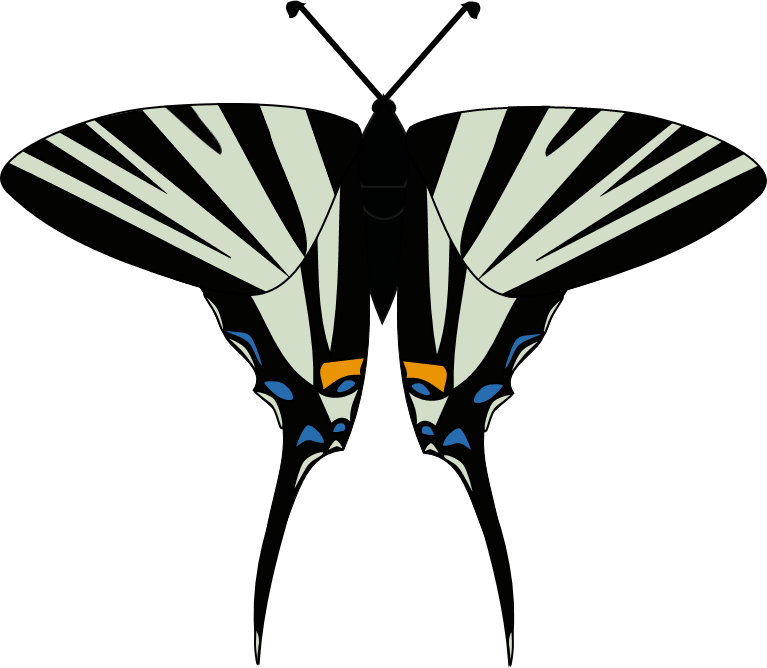
*

**Fig 12. Two *Iphiclides podalirius*** **drawings.** Two *Iphiclides podalirius* drawings with different wing tails length (A) natural aspect (B) modified with 20% longer wing tails.

**Butterfly species:** *Charaxes jasius*

(A)*
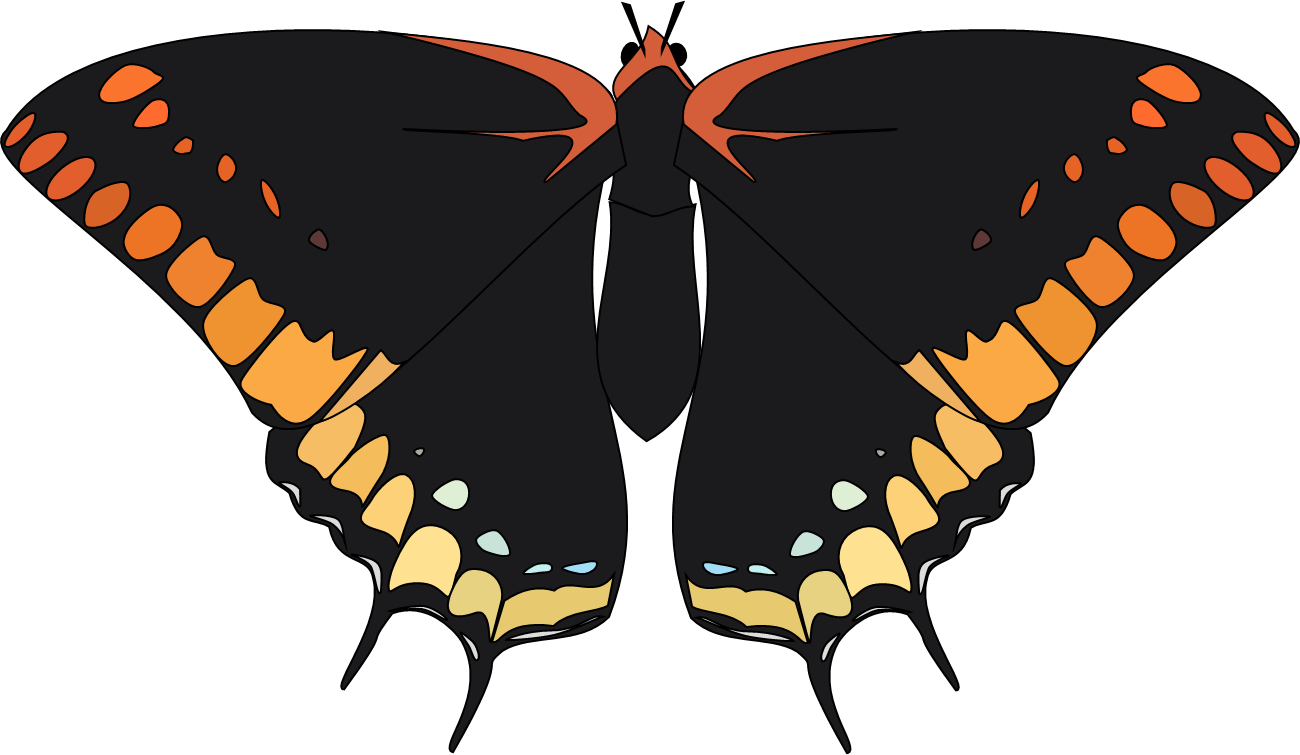
* (B)*
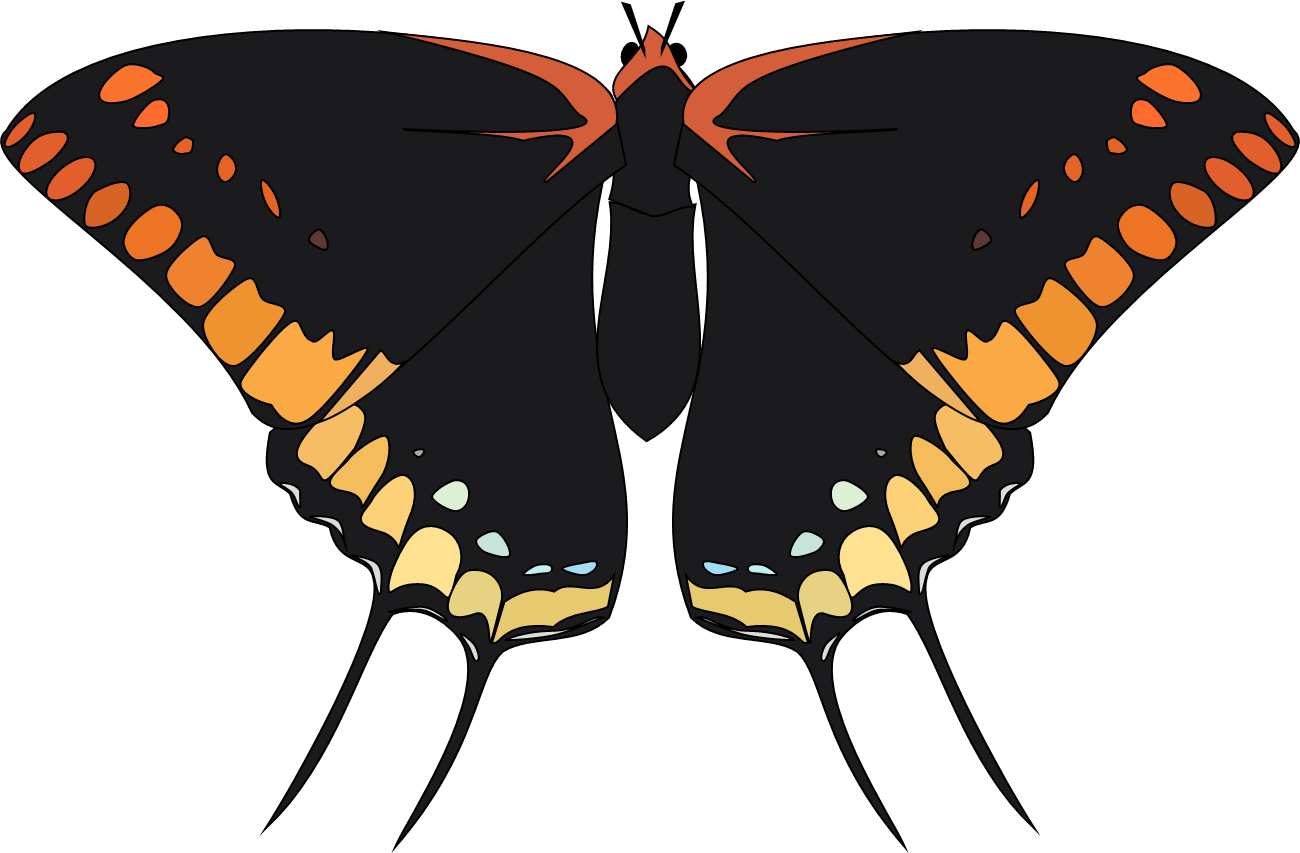
*

**Fig 12. Two *Charaxes jasius*** **drawings.** Two *Charaxes jasius* drawings with different wing tails length (A) natural aspect (B) modified with 15% longer wing tails.

**Morphological feature:** smooth or jagged wing edges

**Butterfly species:** *Polygonia c-album*

(A)
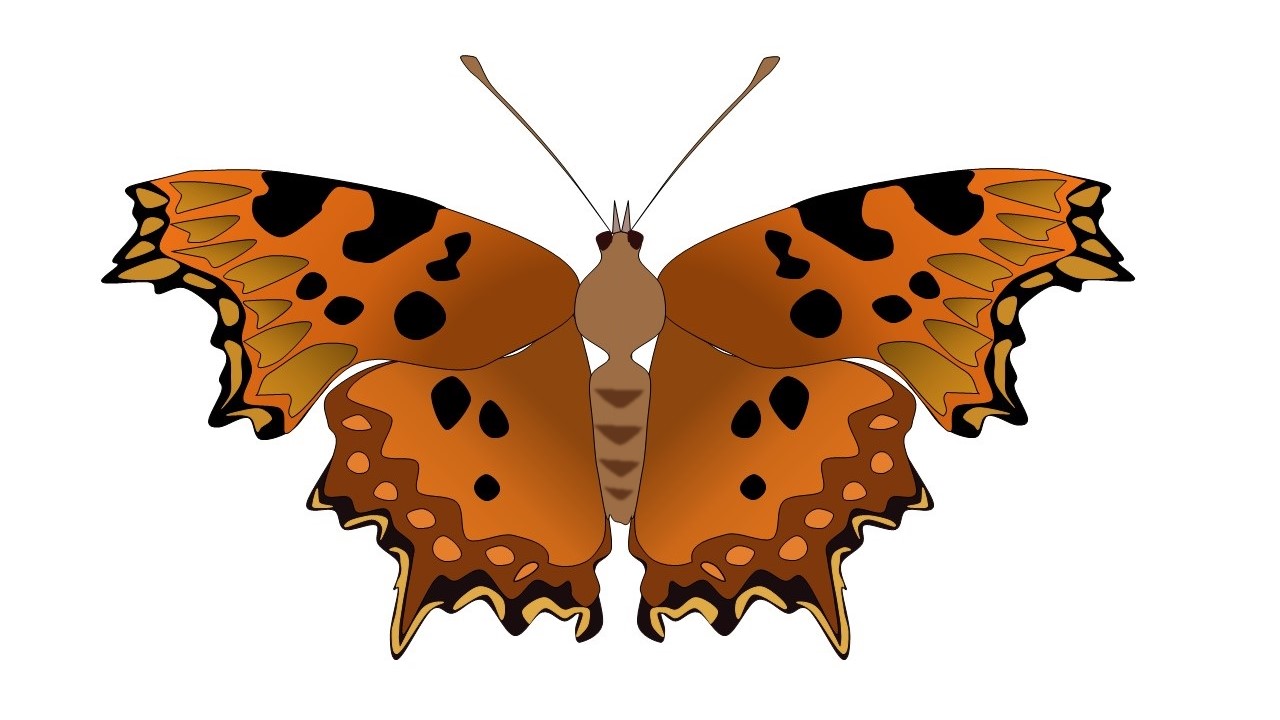
(B)*
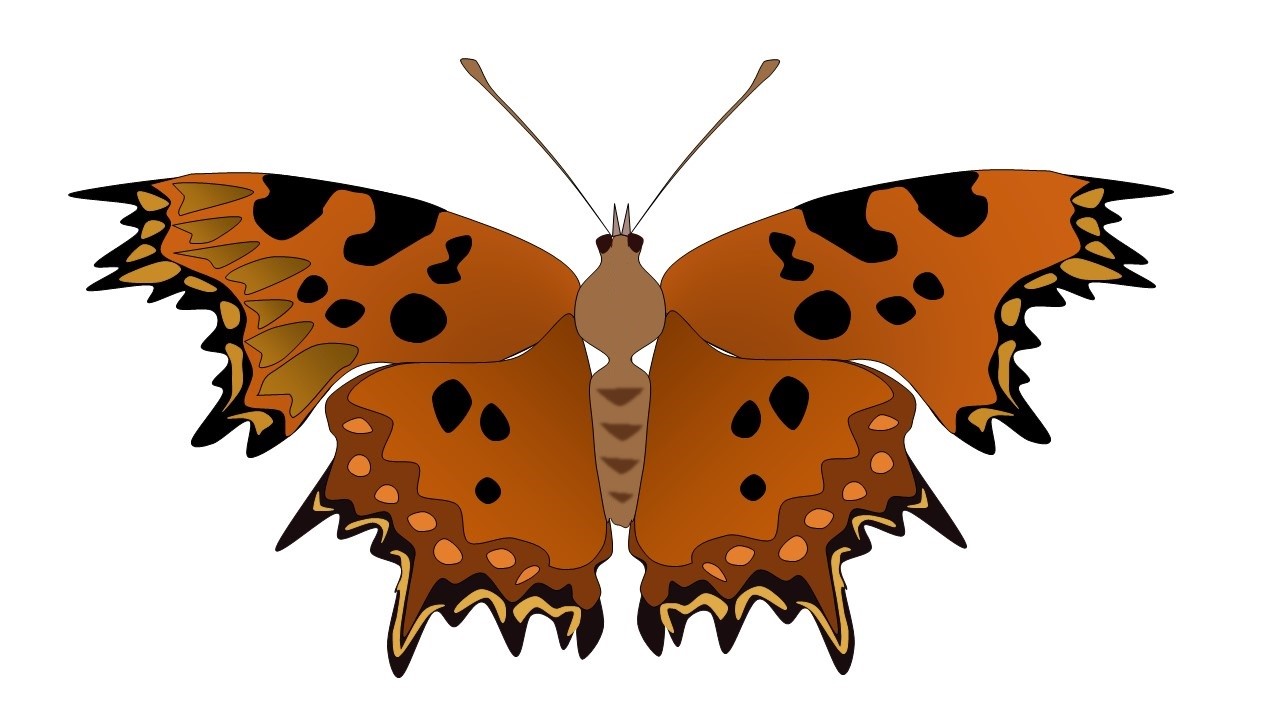
*

(C)*
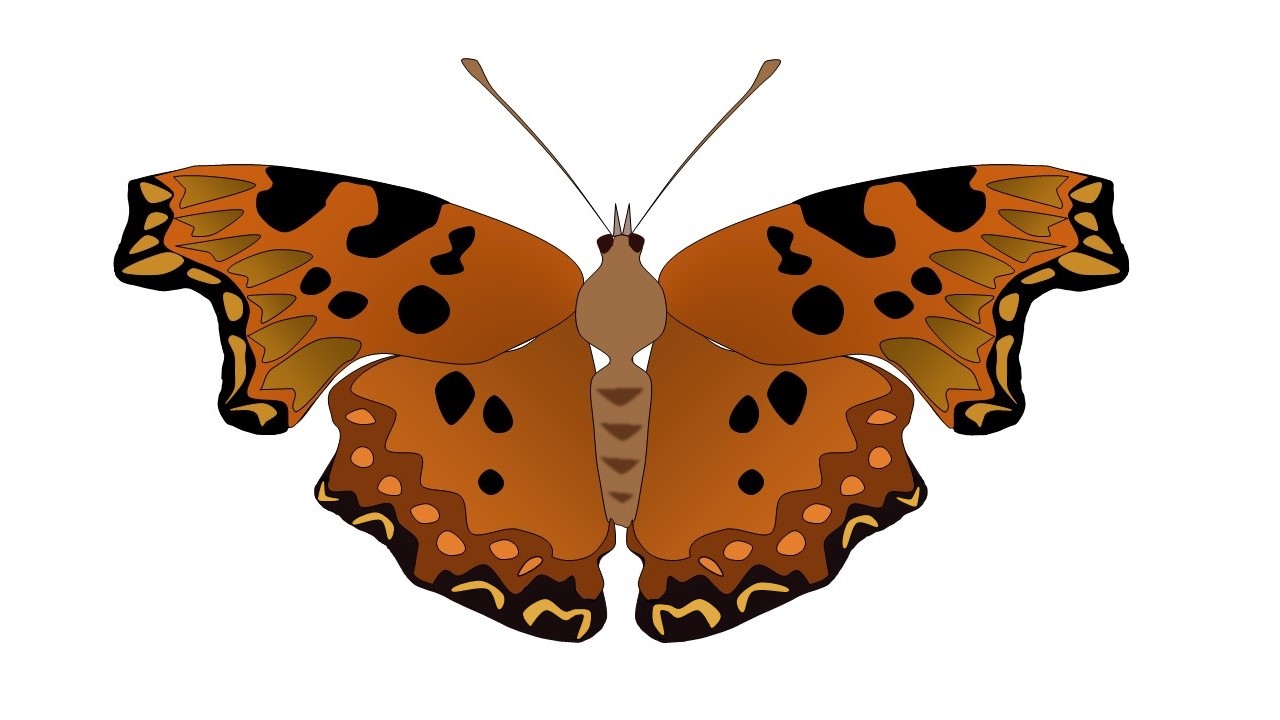
*

**Fig 13. Three *Polygonia c-album* drawings.** Three *Polygonia c-album* drawings with smooth or jagged wing edges (A) natural aspect (B) modified with jagged edges (C) modified with smooth edges.
