# Supplementary material for: Assessing the aesthetic attractivity of European butterflies: A web-based survey protocol
Source: PLoS One. 2023 May 11;18(5):e0283360. doi: 10.1371/journal.pone.0283360 (PMC10174575; doi:10.1371/journal.pone.0283360)
Supplement: Attachment — Submitted filename: Response to reviewers.docx [file pone.0283360.s004.docx]

**Response to Reviewers**

*Original text in black, answers in red.*

**Reviewer #2**: I checked the revised version of the manuscript. In my opinion, all comments and remarks raised in my initial review have been scrupulously addressed by the authors. Besides the detailed replies, the expected corrections are made in the manuscript. I also went through the comments from other reviewers and, also in this case, most of the corresponding amendments have been done. Considering the above I recommend accepting the manuscript and qualifying it for the next stages of processing.

Thank you very much for this kind comment, your findings have been valuable in helping us clarify complex passages which ended up improving our work: we are grateful to you for this.

**Reviewer #4**: Assessing the aesthetic attractivity of European butterflies: a web-based survey protocol

Thank you for sending the manuscript for review. Conservation of endangered species is an ever-present topic that is studied from different perspectives. Aesthetic preferences towards animals are a frequently mentioned factor that plays an important role in the planning and implementation of conservation projects in recent years. These projects, including rescue breeding and zoos, cannot do without public support. Finding out which species are positively perceived by people and why therefore provides valuable insights for the future direction of conservation activities. In recent years, attention has been shifting from the charismatic vertebrate groups to less popular groups and also to invertebrates. The focus of the study on butterflies is an appropriate continuation of similar research. While I appreciate the quality of the submitted project, I have several comments and additional questions on the submitted text.

I missed the zoological definition of the study at the beginning of the paper, i.e. which specific taxa your study is concerned with. The specific species are listed in the Appendix, however, a brief mention in the text would have been appropriate.

Thank you for your comments and suggestions throughout this review. We agree with the lack of such definition, and we modified the protocol accordingly, now reporting it at lines 56-57.

I have further comments on the description of the methodology, which is unclear in some parts. I do not want to criticise your proposed procedure, I just find some information missing in your description.

In the introduction of the methodology, the reference to the summary of the questions used is marked Appendix S3, but Appendix S1 and S2 are mentioned later in the text. It would be more appropriate to change the numbering.

Thank you for pointing this out, we rearranged the Appendixes’ numbering to reflect the order in which they are cited in the protocol (lines 106, 153, 195, 202, 772, 774, 776).

Section n.2: Ranking

As I understand it, each respondent ranked 9 randomly selected images. Were the butterfly pictures presented one at a time or all at once? Alternatively, was a preview of all the ranked images shown first?

All 9 pictures are displayed at once, then when the user clicks on each picture to rank it, they see that picture enlarged (covering the other previews) with a clickable 0-10 meter to express their choice. Nevertheless, we agree with your point and updated the first image on fig. 1 with the actual sight presented to the user, displaying the 9 previews altogether. We also specified this in the manuscript, at lines 140-142.

The procedure for selecting images for the dataset is described and detailed in the appendix, however I would appreciate information on how many images in total were used for this section. As there are not the same number of images from each species, it is not possible to simply count.

The total number of images is: 2288 items. We added it in the appendix.

Section n. 3: Single morphological features

In this section, it is not clear to me what question respondents were asked to choose from a pair of species. Was it the same as in the previous section?

In this section we asked the participants to express a simple preference between two modified images (from a set of images described in S3 Appendix). We now specified the procedure at line 176.

Again, I am missing information on the total number of images tested in this section. There is also no indication of how the drawings of the unmanipulated butterflies were produced or whether they were from any particular source. It would also be useful to indicate what the specific species were selected on. For example, for the analysis of the presence of eye spots, a species that has them present was selected. But were any other parameters taken into account, such as wing colour or shape?

The total number of images displayed in this section is 23 and both the drawings of altered and unaltered species were produced using MS PowerPoint. As starting models, we used pictures of real butterflies. Each species selected was chosen because of the perspicuity of their most salient features (e.g. the dimension of eye spots for *Aglais io*) which, in turn, were chosen according to the referenced entries of scientific literature in experimental aesthetics. No additional parameter other than the most salient feature of each given species was taken into account.

Section n. 4: Emotional engagement

This section details all the species included. However, it is not entirely clear on what basis these species were selected. The last sentence of this section suggests that this was to create a representative sample, but it would have been more appropriate to state this at the beginning. Nevertheless, it should be better explained what the criteria for selecting species were, for example whether the species selected were those that are most abundant in the group or whether this was based on previous studies.

We agree with the opportunity to mention the purpose of the selection at the beginning, rather than at the end of this section, therefore we revised the protocol at lines 217-221 also explaining better the criteria we took into account for the species selection. We thank you for highlighting this deficiency, as it has made possible to fill a significant gap.

Your manuscript is a study protocol. It begs the question, why are you publishing your research at this stage? Your study is very well designed and promises interesting results, so your procedure is not entirely clear to me. For example, is this a publication needed to complete the study of one of the authors?

We believe that publishing the protocol at this stage could result in several benefits. Given the scope of the project, keeping track of how we set out its very first step (which is that of data collection: how we built the online survey in its 5 main sections, how we made our choices as to the selection of butterfly species, which key-features we took into account, how we set up the test for public diffusion and so on) should add consistency to the whole dissemination process. Moreover, publishing at this stage can help us involve more people interested in our research, fuel debate and scientific discussion on the method adopted and its aims and return a detailed description of the basic elements on which we are grafting a study that we expect to extend to a broader level, which would be harder to render once we move on to the discussion of the results. Lastly, we entrust PLOS’ study protocol publication’s policy as to the opportunity to give due academic credit to the foundations of our work.

I also have a few observations to discuss. You mention that by educating and approaching some non-preferred animals over a long period of time, you can change people's perception of these species. In the case of aesthetic preference, that doesn't seem quite possible to me. If a person likes a blue butterfly because they like the color blue, you can't "force" them to like a red butterfly through education. We can make people aware that the red butterfly exists and that it is important to nature/biodiversity/the planet, but we can't change their personal preference for the color blue. Educational programs should teach people that there are animals they may not like, but they are interesting, important and should pay attention to. This may translate into increased attention when visiting a zoo or deciding whether to donate to a conservation program. But it doesn't change the fact that a person likes a blue butterfly.

Thanks for this observation. The overall aim of the study does not concern the chance to modify people’s tastes, persuading them - on the long run - to alter their appreciation for something: it is rather a matter of broadening the spectrum of what they appreciate and take into account in their aesthetic experiences. To this extent, we adopt a “multi-factorial” notion of aesthetic experience, understood as consisting of perception, emotion, and cognitive information (which is the purpose of the different sections making up the test). As several pieces of research (See: Franklin, M. B., Becklen, R. C., Doyle, C. L., 1993: “The Influence of Titles on How Paintings Are Seen”, *Leonardo* 26(2), 103–108. <https://doi.org/10.2307/1575894>; Nodine, C. F., Locher, P. J., Krupinski, E. A., 1993: “The Role of Formal Art Training on Perception and Aesthetic Judgment of Art Compositions”, *Leonardo*, 26(3), 219–227. <https://doi.org/10.2307/1575815>; Cupchik, G. C., Shereck, L., Spiegel, S., 1994: “The Effects of Textual Information on Artistic Communication”, *Visual Arts Research*, 20(1), 62–78. <http://www.jstor.org/stable/20715819>; Reber, R., Schwarz, N., Winkielman, P., 2004: “Processing Fluency and Aesthetic Pleasure: Is Beauty in the Perceiver’s Processing Experience?”, *Personality and Social Psychology Review*, 8(4), 364–382. <https://doi.org/10.1207/s15327957pspr0804_3>; Leder, H., 2001: “Determinants of Preference: When do we like What we Know?”, *Empirical Studies of the Arts* 19(2), 201–211. <https://doi.org/10.2190/5TAE-E5CV-XJAL-3885>; Leder, H., Carbon C.C., Ripsas, A-I., 2006: “Entitling art: Influence of title information on understanding and appreciation of paintings”, *Acta Psychologica* 121 (2), 176-198.; Mari, E.; Quaglieri, A.; Lausi, G.; Boccia, M.; Pizzo, A.; Baldi, M.; Barchielli, B.; Burrai, J.; Piccardi, L.; Giannini, A.M. Fostering the Aesthetic Pleasure, 2021: “The Effect of Verbal Description on Aesthetic Appreciation of Ambiguous and Unambiguous Artworks”, *Behav. Sci.* 11, 144. <https://doi.org/10.3390/bs11110144>) show, what we know (about an object or a living being) affects the kind of emotional connection we establish with it and modifies (at least to a certain extent) the way it appears to us. Therefore, the overall aim of the study is not to “force” people to change their perception – under the important premise that basic perceptual data are not the only component of the aesthetic. It is rather a matter of broadening the spectrum of what we like and of the aesthetic categories that we apply to the natural world (not just “beautiful” and “pleasurable”, but also “sublime”, “eerie”, “surprising”, “diverse”, “magnificent” etc); it is a matter of becoming aware of our (often implicit) preferences and biases and, by means of knowledge and increasing expertise, to mold and shape them in order to make them broader and more inclusive.

I hope my comments help improve your manuscript and I wish you the best of luck with your future research.

They surely did and we are grateful for these important inputs. Thank you for your dedication and good wishes.
